# Biomarkers of chronic liver disease and their determinants in northern Ethiopia: Evaluating the synergistic impact of HBV and Schistosoma mansoni and the contribution of metabolic and lifestyle factors to liver injury

**DOI:** 10.1371/journal.pone.0352266

**Published:** 2026-06-22

**Authors:** Gessessew Bugssa, Nega Berhe, Girmay Medhin, Shevanti Nayagam, Tilahun Teklehaymanot, Asgeir Johannessen

**Affiliations:** 1 Faculty of Medical Laboratory Sciences, Ayder Comprehensive Specialized Hospital and College of Health Sciences, Mekelle University, Mekelle, Ethiopia; 2 Aklilu Lemma Institute of Pathobiology, Addis Ababa University, Addis Ababa, Ethiopia; 3 Department of Microbiology, Immunology and Parasitology, School of Medicine; College of Health Sciences, Addis Ababa University, Ethiopia; 4 Vestfold Hospital, Department of Infectious Diseases, Tønsberg, Norway; 5 Imperial College, London, United Kingdom; 6 University of Oslo, Institute of Clinical Medicine, Oslo, Norway; Kwame Nkrumah University of Science and Technology, GHANA

## Abstract

**Background:**

Liver diseases are a major global health concern, affecting over 1.5 billion people and ranking among the leading causes of death. Sub-Saharan Africa carries a high burden, yet the underlying etiology of chronic liver disease is poorly described. The aim of this study was to assess biomarkers of chronic liver disease and their determinants in Northern Ethiopia.

**Methods:**

A community-based cross-sectional study was conducted between December 2019 and June 2020 among randomly selected participants aged 5 years or older in Alamata district of Tigray region. Socio-demographic, behavioral, and clinical factors were collected via structured questionnaires; blood was tested for liver function biomarkers and Hepatitis B Virus (HBV) and stool samples for *S.mansoni* infection. Liver function biomarkers’ abnormalities were defined based on age and sex-specific thresholds. Multivariable hierarchical logistic regression analysis was performed to identify predictors of abnormal alanine aminotransferase (ALT) and adjusted odds ratios (AOR) with 95% confidence intervals (CI) were reported.

**Results:**

A total of 767 participants were included, with a median age of 27 years (5–80 years); 53.1% were female and 67.5% resided in rural areas. The prevalence of HBV and *S.mansoni* infection was 5.9%, and 26.6%, respectively. Overall, 23.5% of participants had abnormal liver function biomarker with 7.3%, 14.2%, 9,4%, and 8.0% had elevated aspartate aminotransferase(AST), alanine aminotransferase (ALT), alkaline phosphatase (ALP), and total bilirubin levels, respectively. Besides, the De Ritis, aspartate to alanine (AAR) ratio revealed a median of 0.96(IQR:0.62–1.74), with 51% (79/155) of the participants presented with an AAR < 1.0, and 21.3% of participants exhibited an AAR > 2.0. Moreover, the R value depicted the patterns of liver injury with 90(52.0%) participants exhibited R-value below 2(cholestatic injury), whereas 44(22.43%), and 39(22.54%) of the participants were found to have R-values between 2 and 5(mixed injury), and R > 5(hepatocellular injury), respectively. The hierarchical modeling depicted that the infectious domain accounted for the largest portion of model variance (change in Nagelkerke R-squared (ΔR^2^) =0.157, p < .001). Accordingly, the model demonstrated that several determinants of hepatocellular injury led by significant synergistic interaction between HBV and *S. mansoni* co-infection conferred a 19.7-fold increase in the odds of elevated ALT (aOR:19.7; 95%CI:5.50, 70.6; p < .001), significantly higher than mono-infections of HBV (aOR:13.7; 95%CI: 5.82, 32.04; p < .001) or *S. mansoni* (aOR:3.4;95% CI:2.5; 5.51, p < .001). Beyond the infectious synergy, khat chewing (aOR:4.1, 95%CI:2.21, 7.58), and diabetes mellitus (aOR:4.01, 95%CI:1.55–10.36); p = .004) were confirmed as significant independent predictors, each associated with a four-fold increase in likelihood of having elevated ALT.

**Conclusion:**

Liver function biomarkers’ abnormalities were common in northern Ethiopia driven by infectious, metabolic and lifestyle factors. The significant synergy between HBV and *S.mansoni* creates a compounded risk that far exceeds the impact of mono-infections, diabetes, or khat chewing. The findings emphasize the need for integrated public health strategies that address both infectious and non-infectious determinants simultaneously to effectively reduce the burden of chronic liver disease in the region.

## Background

Liver diseases pose a major global health crisis, affecting an estimated 1.5 billion people and contributing to significant illness and mortality [1]. Chronic liver disease (CLD) is defined as liver injury persisting for six or more months without complete resolution. Liver disease rates are consistently rising and ranking among the leading causes of death worldwide [2]. The most severe outcomes, cirrhosis and hepatocellular carcinoma (HCC), represent advanced stages of liver damage and are the primary drivers of liver-related deaths [3].

Various factors contribute to liver disease, each with distinct characteristics and risk of disease progression [4]. Globally, the most common causes include viral hepatitis, alcoholic liver disease (ALD), and metabolic dysfunction associated steatotic liver disease (MASLD) often linked to obesity and metabolic syndrome, all of which can lead to cirrhosis, liver failure, and HCC [5]. Other significant contributors include schistosomiasis [6,7] and khat consumption [8,9].

Sub-Saharan Africa (SSA) bears a high burden of liver disease, primarily from endemic viral infections, schistosomiasis, alcohol, hepatotoxic medications, MASLD, and aflatoxin exposure [10]. Chronic liver disease (CLD) represents a significant health burden in Ethiopia, ranking as the 7th leading cause of death and contributing to 24 deaths per 100,000 population in 2019 [11]. Despite this significant burden, a substantial proportion (about 45%) of CLD cases in Ethiopia have an unidentified etiology, largely due to limited diagnostic capacity and comprehensive evaluation [12]. However, there is a striking lack of population-based studies, and hence the true prevalence of liver disease and underlying causes in Ethiopia is largely unknown. The situation is further compounded by the co-occurrence of multiple risk factors, including Hepatitis B Virus (HBV) infection, *S.mansoni* infection, chronic alcohol consumption, khat use and metabolic factors.

In the present study, therefore, we aimed to provide epidemiological data on biomarkers of chronic liver diseases and associated factors in Tigray region of northern Ethiopia. The findings could provide valuable insight into the liver disease burden in the region and its underlying etiology and may be used as baseline for the development of evidence-based public health strategies and preventive measures tailored to the region’s specific needs.

## Methods

### Study design and setting

This community-based cross-sectional study was conducted in Alamata District, part of the southern zone of the Tigray National Regional State in Northern Ethiopia. Administratively, the district includes 14 rural kebeles (smallest administrative units) and one semi-urban area under the rural administrations as well as four kebeles within Alamata town (urban administrations).

The population of the district exceeds 150,000 [13] with most of the population engaged in subsistence agriculture and the minority being in urban areas. The economy is largely agrarian-based with cereal and vegetable growing as well as livestock rearing, while the urban population engages in small-scale trade as well as services.

### Study period and population

Data was collected between December 23, 2019 to June 12, 2020 among individuals aged 5 years and above. A multi-stage sampling technique was employed. First, five clusters (geographic units) were selected randomly out of the six clusters in the district, ensuring inclusion of both urban and rural clusters. Then from each cluster, two kebeles were randomly selected and proportional allocation was applied based on the total population of each cluster and kebele, ensuring representativeness. Within each kebele, a systematic random sampling technique was used to select households. The details of the sampling procedure are described previously [14].

Since this study was part of a larger project, a subset of patients was randomly identified from an existing dataset. After determining the required sample size for the current study (n = 777), participants were selected using a simple random sampling technique in Microsoft Excel. Specimens for the selected participants had already been collected as part of the larger parent study.

### Data collection

Data collection encompassed socio-demographic, behavioral, and health-related variables. The information was obtained through interviewer-administered questionnaires during home visits. Medical history variables such as diagnoses of diabetes mellitus were assessed. Besides, behavioral risk factors, including alcohol and khat consumption were assessed using a structured questionnaire adapted from previous study [15]. Participants were asked about the type, amount, frequency, and duration of alcohol and khat use. For alcohol, both commercially produced drinks (beer, wine, spirits) and traditional beverages (tella, tej, areki) were included. Participants estimated the amount consumed using locally familiar containers (e.g., birille, glass, shot). For khat, participants reported the amount (measured in locally recognized bundles), and frequency (days per week).

**Stool sample collection and analysis**: Approximately 2–3 grams of fresh stool was collected from each participant in sterile, leak-proof containers. The stool was then processed using the Kato-Katz technique [16]. The slides were examined microscopically by experienced laboratory technologists.

**Blood sample collection and analysis**: From each participant, 5 ml of venous blood was drawn using sterile vacutainer systems and transferred into plain serum separator tubes. Samples were allowed to clot, then centrifuged at 3000 rpm for 10 minutes to separate serum. The sera were aliquoted into sterile cryovials and transported in cold boxes to College of Health Sciences and Ayder Comprehensive Specialized Hospital, Central Laboratory for analysis.

**HBsAg testing**: Hepatitis B surface antigen (HBsAg) was detected using the VIKIA HBsAg rapid immunochromatographic assay (BioMérieux, Lyon, France). The test results were interpreted according to the manufacturer’s instructions.

**Liver function biomarkers**: Aspartate aminotransferase (AST), alanine aminotransferase (ALT), alkaline phosphatase (ALP), total bilirubin (TBil), direct bilirubin (DBil), and albumin (Alb), were performed using the Cobas c 311/501 chemistry analyzers (Roche Diagnostics GmbH, Mannheim, Germany).

**Grading and Interpretation of liver function biomarkers**:

a. Liver enzyme elevations were graded according to the American College of Gastroenterology guidelines [17]. AST, ALT and ALP elevations were categorized as: borderline (<2 × ULN (Upper Limit Normal)), mild (2–5 × ULN), moderate (5–15 × ULN), and severe (>15 × ULN). Based on the International Federation of Clinical Chemistry criteria [18], abnormal liver function biomarkers were defined as values exceeding the upper limit of normal: AST > 40 IU/L (male) and >32 IU/L (female); ALT > 41 IU/L (male) and >33 IU/L (female); ALP > 129 IU/L (male), > 104 (female) youth and adults, and >420 IU/L(male) and >300IU/L (female) for those <15 years of age; Alb < 3.5 g/dL; TBil > 1.2 mg/dL, and DBil > 0.2 mg/dL.
b. The aspartate alanine ratio (AST/ALT, also known as De Ritis ratio) which is abbreviated as AAR was calculated to assess potential etiology of liver injury: a ratio <1.0 indicates non-alcoholic hepatocellular damage, while>=2.0 suggests alcoholic liver disease (ALD) [19,20].
c. The R-value (denoted by ‘R’), is defined as the ratio of alanine aminotransferase (ALT) and alkaline phosphatase (ALP) to their upper limit normal (ULN) and is expressed using the formula: [(ALT/ULN of ALT) ÷ (ALP/ULN of ALP)]. This parameter is used to classify liver injury patterns as hepatocellular (R > 5), cholestatic (R < 2), and mixed (2 < R < 5) types based on the recommendations of European Association for the Study of the Liver (EASL) [5].

### Ethics statement

Ethical clearance was granted by the Institutional Review Board of the Aklilu Lemma Institute of Pathobiology, Addis Ababa University (Reference Number ALIPB/IRB/006/2017/18). Further permission was obtained from the Tigray Regional Health Bureau, Alamata Woreda Health Office, and relevant administrative authorities. Written informed consent was obtained from adult participants, while parental or guardian consent along with participants’ assent was secured for those under the age of 18 after explaining the study’s purpose, procedures, and potential risks.

### Inclusivity in global research

Additional information regarding the ethical, cultural, and scientific considerations specific to inclusivity in global research is included in the Supporting Information (S1_checklist).

### Data analysis

Descriptive statistics, including frequencies, percentages, means, medians, and interquartile ranges (IQRs) were computed, with data normality assessed using Shapiro-Wilk test. Besides, a violin plot was used to visualize the distribution of liver function biomarkers. Non-parametric tests (Mann-Whitney U test and Kruskal-Wallis test) were used to compare the difference in medians for liver function biomarkers. Categorical variables were analyzed using Pearson’s chi-square (χ2) test; however, where cell frequency assumptions were violated (i.e., more than 20% of cells had an expected count less than 5), the Fisher-Freeman-Halton exact test was employed using Monte Carlo simulation (10, 000 samples). Prior to multivariable modeling, multicollinearity was assessed with variance inflation factors (VIF) with values between 1 and 5) were considered acceptable and variables were kept in the model. A multivariable hierarchical logistic regression was then constructed to identify independent determinants of elevated ALT. Variables exhibiting a p < .25 in bivariate analysis were entered in to the model in five sequential blocks (demographics, infection group, alcohol, khat, and metabolic domains), while age and sex were maintained as fixed covariates throughout. Model performance was evaluated using the Omnibus (chi-square test,) Nagelkerke Pseudo R^2^, and change in Nagelkerke R-squared (ΔR²) across blocks with Hosmer-Lemeshow test utilized to ensure the final multivariable logistic regression model was a good-fit for the data. Results of the multivariable analysis were reported as adjusted odds ratios (aOR) with 95% confidence intervals (CI), and findings were visualized using a forest plot to illustrate the magnitude of independent and synergistic associations. All analysis was performed using SPSS version 27.0, with statistical significance set at p < .05.

## Results

### Socio-demographic, lifestyle and health related profiles

A total of 767 (98.7%) participants had complete data and sufficient blood samples for analysis and were included in the study. The median age was 27 years (range 5–80), and 407 (53.1%) were females. The largest age group was 5–14 years representing 27.8% of the total. The majority of the participants (67.5%) were from rural areas. Besides, 219 (28.6%) participants self-reported that they currently consume alcohol while 78 (10.1%) participants reported chewing khat. A total of 204 (26.6%) and 45 (5.9%) participants were infected with *S.mansoni* and HBV, respectively; 14 (1.8%) were infected with both HBV and *S.mansoni* (Table 1).

**Table 1:** Socio-demographic lifestyle and health related characteristics of study participants in Alamata district of Tigray region, northern Ethiopia.

Variable	Categories	Frequency (%) N = 767
Sex	Male	360(46.9)
	Female	407 (53.1)
Age	5-14	213(27.8)
	15-24	141(18.4)
	25-34	119(15.5)
	35-44	107(14.0)
	45-54	89(11.6)
	>=55	98(12.8)
Residence	Urban	249 (32.5)
	Rural	518(67.5)
Religion	Orthodox Christian	666 (86.8)
	Muslim	95(12.4)
	Others	6(0.8)
Educational Status	Illiterate	263(34.3)
	1-8	395(51.5)
	9-12	72(9.4)
	College and above	37(4.8)
	Single/underage	319(41.6)
Marital Status	Married	259(33.8)
	Divorced	118(15.4)
	Widowed	71(9.3)
	Student/underage	281(36.6)
	Farmer	163(21.3)
Occupation	Employed	83(10.8)
	Housewife	80(10.4)
	Merchant	79(10.3)
	Daily laborer	39(5.1)
	Job seeker	42 (5.5)
Currently Chewing khat	Yes	78(10.2)
	No	689(89.8)
Frequency of chewing Khat	>= 5 times/week	45(5.9)
	< 3 days/ week	21(2.7)
	< 1 day/week (occasional)	12(1.6)
Currently consuming Alcohol	Yes	219 (28.6)
	No	548(71.4)
Frequency of Alcohol consumption	5-7 days/week	59(7.7)
	1-4 days/week	148(19.3)
	<1 day/week (occasional)	12(1.6)
	None at all	548(71.4)
Type of alcohol	Beer	52(6.8)
	Spirit	7(1.0)
	Homebrew	130(19.0)
	Mix of alcohols	30(3.9)
HBV infection	Yes	45(5.9)
	No	722(94.1)
*S.mansoni* infection	Yes	204(26.6)
	No	563(73.4)
Diabetes mellitus	Yes	26(3.4)
	No	741(96.6)

### Distribution and pattern of liver function biomarkers

A total of 180 (23.5%) subjects had abnormal liver tests. Of these, elevated levels of AST and ALT were observed in 133 (17.3%) and 109 (14.2%) participants, respectively. Abnormal levels of ALP, albumin, total bilirubin and direct bilirubin were detected in 72 (9.4%), 74 (9.6%), 61 (8.0%) and 49 (6.4%) of participants, respectively (Table 2).

**Table 2:** Prevalence of liver function biomarkers abnormalities among the study participants in Alamata district, northern Ethiopia.

Liver function biomarkers	Median (IQR)	Min.	Max	Abnormal N (%)	Grades of abnormal levels				
					Borderline (<2xULN) N (%)	Mild (2–5ULN) N (%)	Moderate (5–15 ULN) N (%	Severe (>15ULN) N (%)	Hyper
AST (U/L)	27(17-34)	3.8	410	133(17.3)	56(42.1)	32(24.1)	45(33.83)	–	–
ALT (U/L)	24(12-33)	4.0	680.0	109(14.2)	24(22.0)	28(25.7)	54(49.5)	3(2.8)	–
ALP (U/L)	81(59-103)	34.0	1110.0	72(9.4)	47(65.3)	20(27.8)	5(6.9)	–	–
TBiL (mg/dl)	.3(.15−.52)	0.07	5.80	61(8.0)	33(54.1)	28(45.9)	–	–	–
DBiL (mg/dL)	.13(.07−.21)	0.00	3.58	49(6.4)	10(20.4)	36(73,5)	3(6.1)	–	–
Albumin (g/dL)	3.9(3.7-4.2)	1.07	5.56	74(9.6) *	15(19.0)	5(6.3)	22(27.9)	31(39.2)	6(7.6)
				5(0.7) $					

NB: the grades for Albumin are: Borderline low (3.0-3.4g/dL), Mild hypoalbuminemia (2.5-2.9g/dL), Moderate Hypoalbuminemia (1.5-2.4g/dL), Severe hypoalbuminemia (<1.5), Hyperalbuminemia (>5.2g/dL), max: maximum, Min: minimum, IQR: Interquartile range, ULN: Upper Limit Normal, *: below normal, $: above normal

**Fig 1:**
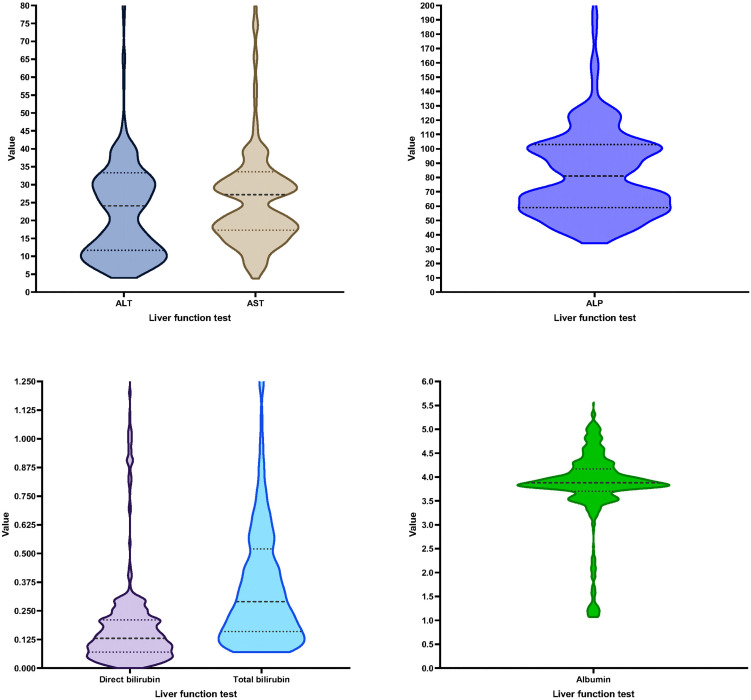
Violin plot showing distribution of biomarkers of liver function (graphs were generated using GraphPad Prism version 10, GraphPad Software, San Diego, CA, USA). Legend: the thick dotted line represents the median, while the thinner dotted lines represent the lower (25%) and, the upper (75%) quartiles).

The biomarkers of liver function were visualized using violin plots, revealing that the majority of values clustered nearly to normal ranges, with relatively few extreme outliers. Specifically, ALT and AST levels were predominantly concentrated in the mid 20s to 30s IU/L, and ALP values clustered between 70 and 90 IU/L. However, direct and total bilirubin levels were heavily skewed toward the lower end of the scale, while albumin levels remained tightly clustered between 3.5 and 4.0 g/dL (Fig 1). Despite this general trend toward normal distribution, an analysis of degree of severity among those with liver function biomarkers’ abnormalities indicated some variations. While most abnormalities were categorized as borderline or mild, ALT stood out as a primary concern, with 57 (52.3%) of abnormal cases reaching moderate or severe elevation. Similarly, 45 (33.83%) of AST and 5 (6.9%) of ALP abnormalities were classified as moderate. Most notably, a concerning pattern emerged in albumin levels; among those with hypoalbuminemia, 31(39.2%) presented with severe depletion, and 22 (27.8%) moderate depletion. In contrast, elevations in direct and total bilirubin were more contained, with 36 (47.4%) and 28 (46.7%) of abnormal results being only mildly elevated, respectively (Table 2).

### Liver function biomarkers by socio-demographic, infectious. lifestyle and metabolic factors

Males had significantly higher median AST (28.2 vs. 23.6, P < 0.001), ALT (26.5 vs. 23.0, P < .001) and ALP (86.0 vs.73.5) levels compared to females. Notably, ALT and ALP levels were higher in the lower age group (5–14 years) (P < .05). HBV positive participants had higher levels of AST, ALT, ALP, Tbil and DBil compared to HBV negative participants. *S. mansoni* infected participants also had higher levels of AST, ALT and Tbil. Of note, khat chewers had significantly higher levels of AST, ALT and ALP, and lower levels of albumin, compared to non-chewers. Participants with diabetes mellitus exhibited significantly higher AST, ALT, ALP and DBil levels compared to those without diabetes mellitus. However, no significant differences were observed with regard to alcohol consumption (Table 3).

**Table 3:** Comparison of median values of liver function biomarkers by socio-demographic, infectious, lifestyle and metabolic factors in Alamata district, northern Ethiopia.

Variables		Liver Function Biomarkers, Median (IQR) values					
		AST	ALT	ALP	TBL	DBL	ALB
Sex	Male(n = 360)	28(18-39)	27(12-39)	86(58-119)	.30(.17−.52)	.13(.07−.21)	3.87(3.70-4.12)
	Female (n = 40	23(17-37)	23(12-39)	74(59-100)	.27(.15−.54)	.13(.06−.21)	3.89(3.75-4.2)
	P-value	<.001	<.001	.001	.443	.604	.198
Age group (in years)	5-14(n = 213)	28 (18 –34)	29(17-39	89(65-109)	.26(.15−.56)	.13(.08−.22)	3.91(3.8-4.3)
	15-24(n = 141)	27 (17 –32)	24(11-32)	71(556−100)	.32(.17−.53)	.13(.07−.21)	3.90(3.8-4.1)
	25-34(n = 119)	22(17-35)	20(11-32)	73(59-104)	.24(.14−.45)	.15(.07−.22)	3.83(3.5-4.1)
	35-44(n = 107)	28(7-40)	19(10-37)	85(55-1102)	.31(.16−.46)	.12(.06−.21)	3.83(3.6-4.1)
	45-54(n = 89)	23(17-35)	17(12-32)	71(59-98)	.31(.17−.56)	.14(.07−.22)	3.90(3.7-4.2)
	>=55(n = 98)	28 (19 –32)	18(11-30)	75(60-104)	.30(.15−.50)	.12(.05−.21)	3.90(3.7-4.2)
	P-value	.879	<.001	.009	.980	.801	.178
HBV infection	Yes (=45)	76(31-216)	88(29-306)	121(91-427)	1.26(.26-2.4)	.26(.14−.91)	3.5(1.7-4.0)
	No (=722)	25 (17 –32)	24(14-32)	74(58-102)	.27(.15−.50	.13(.07−.21)	3.9(3.9-4.2)
	P-value	<.001	<.001	<.001	<.001	<.001	<.001
*S. mansoni* infection	Yes (=204)	29(18-56)	30 (15 –30)	85(59-106)	.35(.18−.68)	.13(.07−.23)	3.9(3.6-4.2)
	No (=563)	25 (17 –32)	21(16-44)	74(59-102)	.26(.15−.47)	.13(.70−.21)	3.9(3.8-4.2)
	P-value	<.001	<0.001	.091	<.001	.795	0.79
Alcohol consumption (frequency)	None	27 (17 –32)	24(12-33)	82(59-103)	.27(.15−.51)	.13(.07−.21)	3.9(3.7-4.2)
	<1 day/week	30(16-69)	29(19-67)	93(52-109)	.28(.15−.78)	.06(.03−.14)	3.8(3.8-4.2)
	1-4 days/week	28(17-37)	23(11-32)	69(56-102)	.28(.16−.52)	.14(.08−.22)	3.9(3.6-4.1)
	5-7/week	30(18-75)	28(11-83)	90(67-106)	.36(.19−.66)	.15(.07−.21)	3.9(3.6-4.3)
	P-value	0.053	.292	0.098	.318	.264	.852
Khat chewing	Yes (78)	33(19-70)	34(14-218)	92(65-152)	.34(.17−.59)	.14(.06−.21)	3.8(3.6-4.0)
	No (=689)	27(17-	23(11-32)	76(58-102)	.27(.16−.52)	.13(.07−.21)	3.9(3.3-4.2)
	P-value	33)<0.001	<.001	0.0208	.171	.958	0.022
Diabetes mellitus	Yes(n = 26)	70(19-207)	76(16-217)	109(55-170)	.39(.11-1.70)	.16(.1−.9)	3.21(1.3-3.9)
	No (=741)	27 (17 –33)	24(11-33)	76(59-103)	.27(.16−.52))	.13(.07−.21)	3.9(3.8-4.2)
	P-value	<.0001	<.001	.023	.072	.043	<.001

IQR: interquartile range, AST: amino aspartate transferase; ALT: alanine aminotransferase, ALP: alkaline phosphatase; TBL: total bilirubin; DBL: Direct bilirubin; ALB: albumin

Furthermore, the cross-tabulation analysis shown in Table 4 indicated that males had a significantly higher prevalence of elevated (>ULN) ALT levels compared to females (18.8% vs. 9.8%, p < .001). Age and alcohol consumption were not significantly associated with elevated ALT, whereas infectious and metabolic factors demonstrated strong associations. In comparison, elevated ALT was observed in 55.6%, and 25.0% of individuals with HBV, and *S. mansoni* infections, respectively. Notably, among the small subgroup of individuals with HBV and *S. mansoni* co-infection, 64.3% (9/14) exhibited elevated ALT levels (p < .001). Similarly, significant associations were also observed for khat chewing and diabetes mellitus (P < .001).

**Table 4:** Distribution of ALT levels relative to the upper limit of normal (ULN) by socio-demographic, infectious, lifestyle and metabolic factors in Alamata district, northern Ethiopia.

Predictors		ALT		
		Normal N (%)	>ULN N (%)	P-value
Sex	Male	298(81.2)	69(18.8)	<.001
	Female	361(90.3)	39(9.8)	
Age category (in years)	5-14	182(85.4)	31(14.6)	.224
	15-24	124(87.9)	17(12.1)	
	25-34	100(84.0)	19(16.0)	
	35-44	85(79.4)	22(20.6)	
	45-54	80(89.9)	9(10.1)	
	>=55	88(89.8)	10(10.2)	
HBV infection	Yes	20(44.4)	25(55.6)	<.001
	No	639(88.5)	83(11.5)	
*S. mansoni* infection	Yes	153(75.0)	51(25.0)	<.001
	No	506(89.9)	57(10.1)	
*HBV & S. mansoni* co-infection	Yes	5(35.7)	9(64.3)	<.001
	No	654(86.9)	99(13.1)	
Alcohol consumption	Yes	184(84.0)	35(16.0)	.339
	No	475(86.7)	73(13.3)	
Khat chewing	Yes	49(62.8)	29(37.2)	<.001
	No	610(88.5)	79(11.5)	
Diabetes mellitus	Yes	14(53.8)	12(46.2)	<.001
	No	645(86.9)	96(13.0)	

ALT: Alanine amino transferase, ULN: Upper Limit Normal

To further elucidate the specific nature of hepatic damage, derived indices including the De Ritis ratio (AST/ALT) and the R-value were computed. To determine the etiologies of liver injury among participants presenting with elevated transaminases, the AST/ALT ratio (AAR) was determined. Accordingly, the median AAR was 0.96(IQR:0.62–1.74), with 51% (79/155) of the participants presented with an AAR < 1.0, while 27.7% % fell between 1.0 and 2.0 AAR range. Additionally, 21.3% exhibited an AAR > 2.0. Besides, a chi-square test of independence revealed that HBV positivity was significantly associated with AAR (χ2 = 54.10, P < .001) with post-hoc analysis showing that HBV positive individuals were significantly over-represented in the AAR category below 1.0 (suggestive of acute hepatocellular injury (adjusted residual = 7.3). Similarly, alcohol consumption frequency also showed a significant relationship (χ2 = 18.42, P < .05). Frequent drinkers (5–7 days per week) predominantly exhibited an AAR > 2.0(suggestive of alcoholic hepatitis), whereas non-drinkers were more likely to have an AAR < 1.0(suggestive of non-alcoholic etiologies) (Table 5).

**Table 5:** Distribution of AST/ALT ratio by socio-demographic, infectious, lifestyle and metabolic characteristics in Alamata district, northern Ethiopia.

Variables		AAR values				P-value, χ2
		<1.00 N(%)	1.00-1.499 N(%)	1.500-1.99 N(%)	>=2.00 N(%)	
Sex	Male	46(58.2)	13(44.8)	6(42.9)	16(48.5)	.410
	Female	33(41.8)	16(55.2)	8(57.1)	17(51.5)	
Age group	5-14	25(31.6)	6(20.7)	5(35.7)	37(17.4)	.793
	15-24	17(21.5)	3(10.3)	2(14.3)	33(23.4)	
	25-34	12(15.2)	7(24.1)	2(14.3)	30(25.2)	
	35-44	12(15.2)	7(24.1)	0(0.0)	30(28.0)	
	45-54	8(10.1)	2(6.9)	3(21.4)	23(25.8)	
	>=55	5(6.3)	4(13.8)	3(21.4)	28(28.6)	
Occupation	Employed	4(5.1)	3(10.3)	3(21.4)	1(3.0)	.240
	Merchant	9(11.4)	4(13.8)	0(0.0)	1(3.0)	
	Farmer	21(26.6)	7(24.1)	3(21.4)	10(30.3)	
	Housewife	4(5.1)	2(6.9)	3(21.4)	4(12.1)	
	Daily worker	2(2.5)	0(0.0)	0(0.0)	3(9.1)	
	Student	34(43.0)	9(31.0)	4(28.6)	13(39.4)	
	Job seeker	5(6.3)	4(13.8)	1(7.1)	1(3.0)	
HBV infection	yes	19(24.1) **	6(20.7)	2(14.3)	5(15.2)	<.001, 63.5
	no	60(75.9)	23(79.3)	12(85.7)	28(84.8)	
*S. mansoni* infection	Yes	39(49.4) **	10(34.5)	6(42.9)	14(42.4) *	<.001, 32.5
	No	40(50.6)	19(65.5)	8(57.1)	19(57.6)	
Khat	yes	16(20.3) **	9(31.0) **	3(21.4)	5(15.2)	<.001, 26.9
	no	63(79.7)	20(69.0)	11(78.6)	28(84.8)	
Alcohol consumption (frequency)	Not at all	47(59.5)	18(62.1)	10(71.4)	22(66.7)	.015, 22.6
	<1 in a week	2(2.5)	1(3.4)	1(7.1)	0(0.0)	
	1-4 days	19(24.1)	8(27.6)	2(14.3)	4(12.1)	
	5-7 days a week	11(13.9)	2(6.9)	1(7.1)	7(21.2) **	
Diabetes status	yes	7(8.9) *	3(10.3) *	2(14.3)	3(9.1)	001, 22.5
	no	72(91.1)	26(89.7%)	12(85.7)	30(90.9)	

Note: * (Adjusted Residual > 1.96); ** (Adjusted Residual > 2.58)

Moreover, to characterize the biochemical features associated with different patterns of liver injury, the R-value was determined for those who had abnormal or elevated AST, ALT, and/or ALP. Accordingly, the median R-value was found to be 1.97(IQR = 0.83–4.36) with 90(52.0%) of participants having R-value below 2 (cholestatic liver injury) followed by 44(25.43%) with R-value between 2 and 5 (mixed type liver injury), and 39(22.54%) having R-values >5 (hepatocellular liver injury) patterns.

To this end, the cross-tabulation of R-value with De Ritis ratio reveals a clear spectrum of pathologic patterns. The hepatocellular group(R > 5) showed significantly higher frequency of acute injury markers, with 34(85.0%) of participants exhibiting a De Ritis ratio below 1.0 suggesting that this pattern is characterized by a more pronounced ALT elevations relative to AST with likely diagnosis of acute viral hepatitis. Conversely, as the R-value decreased toward a cholestatic pattern (R < 2), the proportion of De Ritis ratios increased with 9(33.3%) of these participants presented a ratio >1 suggesting biliary obstruction or cirrhosis compared to only 2.5% in the hepatocellular group. On the other hand, 28(62.8%) of the participants in the mixed type pattern(2 < R < 5) presented with AAR of <1 suggesting non-alcoholic hepatitis such as NAFLD (Table 6).

**Table 6:** Cross tabulation of R value and AST/ALT ratio showing the frequency and pattern of liver disease in Alamata district, northern Ethiopia.

R value	AST/ALT ratio	Frequency (%)	Likely diagnosis (Pattern of diseases)
>5(Hepatocellular)	>2	1(2.5%)	Alcoholic hepatitis
>5 (hepatocellular)	<1	34(85.0%) *	Acute viral hepatitis/NAFLD
<2(cholestatic	>1.0	9(33.3%)	Biliary obstruction/Cirrhosis
2-5(mixed)	<1	28(62.8%)	Chronic hepatitis/NASH

*P-value < .05(with Adjusted residual of 5.2), NAFLD: non-alcoholic fatty liver disease, NASH: non-alcoholic steatohepatitis.

### Synergistic and independent predictors of elevated ALT

To evaluate the independent determinants of hepatocellular injury, a multivariable hierarchical logistic regression model was constructed using elevated ALT as the primary indicator. Following a bivariate screening (p < .25), age and sex were retained as fixed covariates in the first block to ensure robust adjustment of the reported odds ratios. The hierarchical analysis revealed that the infectious domain (Block 2) was the primary driver of liver injury, contributing to significant increase in explanatory power (ΔR^2^ = 0.157, p < .001) and explaining an additional 15.7% of the variance beyond the demographic baseline (Table 7).

**Table 7:** Model summary for hierarchical logistic regression.

Block	Variable domain	Omnibus χ2 (p)	Nagelkerke R^2^	ΔR^2^
1	Demographics	19.2 (P = .004)	0.044	.044
2	Infection Groups	71.9(P < .001)	.201	.157
3	Alcohol	.033(P = .856)	.201	.00
4	Khat	16.94(P < .001	.236	.035
5	Metabolic (Diabetes mellitus)	7.62 (P = .006)	.252	.016

Note: χ²: chi-square, R²: Nagelkerke Pseudo R-squared, ΔR²: change in Nagelkerke R-squared, p: statistical significance. Block 1 includes age and sex as fixed covariates.

**Fig 2:**
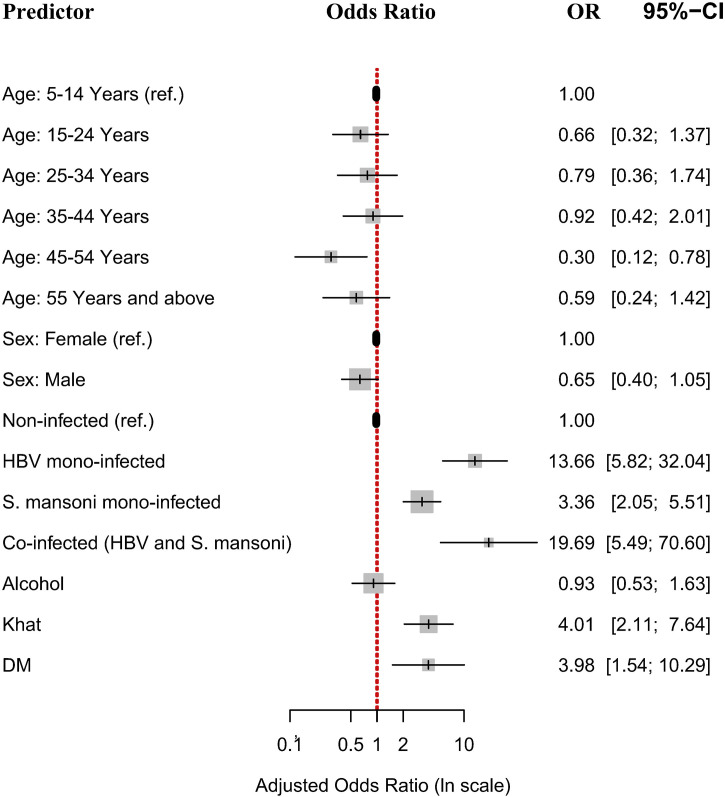
Forest plot representing the final multivariable hierarchical logistic regression model for elevated ALT. Legend: The X-axis is presented on a natural log (ln) scale; data points represent the adjusted odds ratios (OR), and error bars represent the 95% confidence intervals (CI); and the vertical dashed line indicates the null effect (OR=1.0).

Results showed that the 45–54 age group had 70% lower odds of abnormal ALT compared to the 5–14 age group (aOR:0.30, 95%CI:0.12, 0.78; p = .014). To this end, a profound synergistic association was observed among co-infected individuals; those with dual HBV and *S. mansoni* infection were 19.7 times more likely to exhibit elevated ALT (aOR:19.7; 95% CI: 5.50–70.6; p < .001) a magnitude substantially higher than that observed for HBV (aOR:13.7) or *S. mansoni* (aOR:3.4) mono-infections. While metabolic and lifestyle factors were also confirmed as independent determinants, with khat chewing (aOR:4.1, p < .001) and diabetes mellitus (aOR: 4.01, p = .004) each associated with a four-fold increase in the likely hood of having elevated ALT, their effect sizes were notably smaller than the infectious synergy. As visualized in the forest plot (Fig 2), the clear separation of the co-infection estimates from the single-infection domains provides compelling visual evidence of a synergistic interaction where the combined burden of pathogens significantly amplifies odds of having liver injury beyond the sum of their individual effects.

## Discussion

Our findings provide valuable insights in to the prevalence of liver function biomarkers abnormalities and associated socio-demographic, behavioral, and health-related factors in the Alamata District of the Tigray region, Northern Ethiopia.

Our study found that a notable proportion of participants (23.5%) exhibited one or more abnormal liver function biomarker, with 14.2% having an elevated ALT which is the most specific marker of liver cell injury, suggesting considerable burden of liver-related morbidity in the region. The results are consistent with a study conducted in Gonder in northwest Ethiopia [21], but higher compared to reports from other parts of the world [22,23], which could be ascribed to geographic and methodological differences.

Males exhibited significantly higher serum ALT and AST levels compared to females, which is consistent with studies from Taiwan and the United states [24,25]. Men are known to have more severe liver disease than women of almost any cause, including HBV, and consume more alcohol than women [26]. Moreover, it was also recognized that males had higher ALP levels than females. This is consistent with a study conducted in a Spanish population where males generally have higher ALP levels than females during adulthood though this trend is reversed in women after menopause due to increased bone turnover [27]. Similarly, serum ALP levels also varied significantly across age groups. Participants in the 5–14 years age group exhibited elevated ALP levels compared to Adults, a finding that must be interpreted with caution in the context of chronic liver disease. In pediatric populations, high ALP is frequently a physiological manifestation of intense osteoblastic activity during normal bone growth rather than a definitive marker of hepatic pathology [28,29]. Previous studies have documented that serum ALP levels fluctuate in alignment with pubertal stages, typically peaking between 10 and 15 years of age [27] corresponding with the findings of this study. Therefore, while ALP remains a component of the liver function biomarker, its diagnostic utility for liver injury in this specific age is limited unless accompanied by other cholestatic markers which are not influenced by bone metabolism. Contrary to the usual trends where ALT levels usually rise with age [30–32], this study revealed a higher prevalence of elevated ALT in the younger age (5–14 years) group. Though this seems atypical from a physiological standpoint, such discrepancy could stem from the region’s heavy burden of infectious diseases. Children in these areas often encounter *S.mansoni* during routine water-contact activities, causing early liver damage [33]. Moreover, HBV’s progression frequently keeps children in an active immune phase, driving up ALT levels beyond those in older, inactive carriers [34].

In this study, elevated ALT levels were significantly associated with HBV infection, with more than half of HBV positive individuals having an ALT > ULN, in line with reports from Ethiopia [35] and other HBV-endemic settings such as Sudan [36], Nigeria [37], and Pakistan [38]. The high correlation between HBV and elevated ALT emphasizes viral hepatitis as a primary driver of liver injury in this community. This aligns with the natural history of HBV in endemic regions, where early life infection frequently results in prolonged immune activity and significant liver damage [39,40]. The strong association observed in this study underscores the urgent need for expanded screening and antiviral treatment to mitigate these risks. Notably, nearly half of individuals with elevated ALT had *S.mansoni* infection, a well-established cause of periportal fibrosis in endemic regions [41,42]. In line with our findings, a study from northeast Ethiopia [7] documented the impact of *S.mansoni* infection on various liver function parameters. A review of hepatosplenic schistosomiasis reported substantial increase of key liver injury markers such as ALT, AST, ALP, and bilirubin among infected individuals compared with healthy controls [43]. Our study underscores the contribution of this parasitic infection to hepatic injury in endemic regions.

Moreover, the findings of this study demonstrated that the synergistic association between HBV and *S. mansoni* creates a compounded impact on liver health, with co-infected individuals facing a significantly higher odds of liver injury compared to those with mono-infections. These results are consistent with a recent meta-analysis study, which found that schistosomal co-infections with hepatitis B had were associated with approximately six times higher odds of developing liver fibrosis or cirrhosis [44]. This suggests that viral replication and parasitic inflammation concurrently exacerbate liver damage beyond their individual effects. Such phenomenon is frequently encountered in endemic regions such as Ethiopia and Egypt where the overlapping burden of these pathogens accelerates the progression of liver scarring and worsens overall hepatic pathology [45].

Interestingly, the proportion of abnormal ALT was also higher among individuals with self-reported diabetes. Previous studies have showed the association of diabetes mellitus, steatotic liver disease and elevated levels of liver enzymes particularly ALT [46–48]. In type 2 diabetes, insulin resistance leads to increased glucose production and fat accumulation in the liver, causing enzymes to leak into the bloodstream, which results in the elevated liver enzyme levels [46]. An increased burden of steatotic liver disease has been observed over the past decades, in parallel with the global obesity epidemic, and its contribution to the burden of liver disease is predicted to rise also in Africa [49].

Khat chewing was significantly associated with abnormal levels of AST, ALT, ALP, and albumin. Importantly, this study revealed that around one third of khat chewers had abnormal ALT levels. Although the mechanisms are not clear, previous studies from Ethiopia [50], Yemen [51], and Somaliland [52] have suggested that regular khat use can lead to abnormal liver function biomarkers and acute and chronic liver disease. Our study contributes to the growing body of evidence on the harmful effects of this habit and its potential impact on liver health in this context.

Furthermore, alcohol consumption also showed a characteristic biochemical signature. The AST/ALT (De Ritis) ratio demonstrated that individuals reporting alcohol use predominantly had AST/ALT ratios>2.0, a widely accepted threshold for alcoholic hepatitis, where pyridoxal-5′-phosphate (vitamin B6) deficiency suppresses ALT activity while mitochondrial injury increases AST release [53–55]. Similar patterns have been documented in Uganda, where self-reported alcohol intake was associated with elevated AST/ALT ratios, and about 11% had ratios >2.0 consistent with alcoholic hepatitis [56]. On the other hand, HBV-positive participants more often exhibited an AST/ALT (De Ritis) ratio<1.0, consistent with classical descriptions of acute or immune-active viral hepatitis, in which cytosolic ALT with its longer half-life tends to rise more than AST [20,55,57]. To this end, our R-value analysis (based on ALT and ALP) further indicated that over half of the participants showed a cholestatic pattern (R < 2), suggesting a considerable burden of biliary or mixed hepatobiliary involvement, which may relate to schistosomal periportal changes, drug exposures, or other cholangiopathic processes. When cross-tabulated with the De Ritis ratio, we found that 85.0% of individuals with a predominantly hepatocellular pattern (R > 5) also had AAR < 1.0, reinforcing the value of combining these indices to identify acute parenchymal damage, particularly in viral hepatitis. Nonetheless, prospective studies are warranted to determine how best to integrate AAR, R-value, and individual liver biomarkers into clinically useful algorithms for differentiating and monitoring diverse liver injury patterns in resource-limited, co-endemic settings.

Our study had important strengths, including the community-based sampling, the inclusion of several health and behavioral variables, the large sample size, and the use of standardized laboratory testing. We also acknowledge certain limitations. While liver function biomarkers are useful indicators of hepatic injury, they cannot determine causality. Besides, single-time measurements may reflect transient abnormalities rather than chronic liver disease. Moreover, we only used liver enzyme levels without the use of imaging modalities or fibrosis biomarkers which might have resulted in an underestimation of liver disease. Furthermore, the cross-sectional nature of this study limits causal inference. Finally, reliance on self-reported behavioral factors, such as alcohol and khat use, might have introduced potential reporting bias.

## Conclusion

Approximately one quarter of the study participants exhibited at least one abnormal liver function biomarker, with 14.2% specifically showing elevated ALT, indicating a significant burden of undiagnosed liver disease in the Alamata district. While, HBV, *S.mansoni*, diabetes mellitus, and khat chewing are independent determinants of liver injury, this study further demonstrates a compounded impact from HBV and *S.mansoni* co-infection. Multivariable hierarchical logistic regression confirms that this co-infection significantly increases the likelihood of injury compared to mono-infections. Furthermore, the use of liver biomarker indices in this study provide additional clinical insight in to the nature of chronic liver disease. Overall, the findings underscore the substantial contribution of both infectious and non-infectious factors to the burden of liver disease in this setting, and the need for integrated screening and management strategies to reduce the impact of these conditions on liver health.

## Supporting information

S1_File Excel data showing main findings. (XLSX)

S2_File Questionnaire to assess socio-demographic, behavioral, and clinical characteristics. (PDF)

S3_File Laboratory reporting formal for HBV and *S. mansoni* infections. (PDF)

S4_File Laboratory result reporting format for Biomarkers of Liver function. (PDF)

S1_checklist Inclusivity in global research checklist. (DOCX)
