# Supplementary material for: Biomarkers of chronic liver disease and their determinants in northern Ethiopia: Evaluating the synergistic impact of HBV and Schistosoma mansoni and the contribution of metabolic and lifestyle factors to liver injury
Source: PLoS One. 2026 Jun 22;21(6):e0352266. doi: 10.1371/journal.pone.0352266 (PMC13286152; doi:10.1371/journal.pone.0352266)
Supplement: S2_File — (PDF) [file pone.0352266.s002.pdf]

## I. Consent form

**Study title**: Clinico-Epidemiological Study of Liver Disease and Risk Factors in Northern Ethiopia

**Principal Investigator**: Mr. Gessessew Bugssa Hailu, Aklilu Lemma Institute of Pathobiology, College of Health Sciences, Addis Ababa University

I am fully aware that this research undertaking is supported and coordinated by the Aklilu Lemma Institute of Pathobiology (ALIPB), College of Health Sciences, Addis Ababa University. The Principal Investigator is Mr. Gessessew Bugssa Hailu. I have been fully informed in a language I understand regarding the objectives of this research project.

I have been assured that all information provided to the interviewer will remain strictly confidential. I understand that participation involves no foreseeable risks and no financial compensation. I am also aware of my right to withhold information, skip specific questions, or withdraw from the study at any time without penalty or the need to explain my reasons. I have been informed that refusal to participate will have no effect on my health benefits or any administrative effect.

Should I have any questions or require further information before, during, or after the study, I may contact:

**AAU Aklilu Lemma Institute of Pathobiology IRB Office**: +251112763091

**Principal Investigator**: Gessessew Bugssa Hailu (Tel: +251 913 252 205)

**Principal Advisor**: Dr. Nega Berhe, ALIPB (Tel: +251 911 408 340)

**Consent**: ☐ Yes ☐ No (If yes, continue to the interview, if no please thank and quit)
**Participant Signature**: __________ Date: ________

**Data Collector's name**: _____________________ Signature: _________ Date:_______

## II. Parental Permission

**Study Title**: Clinico-Epidemiological Study of Liver Disease and Risk Factors in Northern Ethiopia

I, (parent/guardian), give my permission for my child to participate in this study. I understand the study's objectives, procedures (questionnaire, give stool sample and, 5ml of blood sample), minimal risks, no compensation, voluntary participation, confidentiality, and the right to withdraw at any time without consequences.

Parent/Guardian Signature: _________ Date: _________

Data Collector’s Name: _____________________ Signature: ______ Date: ______

## III. Assent Form (Children)

Study Title: Clinico-Epidemiological Study of Liver Disease and Risk Factors in Northern Ethiopia

Hi! My name is [----------]. We're doing a study about liver health. Your parent has agreed that you can participate in the study if you want.

***What will happen?***

- I will ask you some questions like your age, sex, education, place of residence, and other questions about your health (10-15 minutes).
- Health professionals will instruct you to bring small amount of stool and they will take a small amount of blood sample to know about the health of your liver.
- I won't use your real name when I write my report

***Do you have to do this?***

No! It's your choice. Even if your parents said" Yes", you can say "No" if you don't want to do. No one will be angry at you if you say no or if you want to stop later.

***Questions?***

- If you don't understand anything you can just ask me what I am doing.
- If there is something you don't understand or are scared about you can talk to your parents.
- You can call later if you need anything related to this study.

I want to be in the study:

☐ Yes ☐ No

Child Signature: ___________ Parent/Guardian signature: ___________ Date: ______

Data Collector's Name: ________________ Signature: ___________ Date: _______

Phone no: +251913252205(Gessessew Bugssa),

# IV. Socio-demographic information and other clinical data

**Instruction:** Please fill the blank space for the open-ended question and encircle the answer for the given responses of the study participants in the question item.

| **1. Socio-demographic information and other clinical questions** | **Responses** | **Remark** |
|---|---|---|
| 1.1. Code | ………………….. | |
| 1.2. Phone number: | ……………………….. | |
| 1.3. Residence (make circle) | 1. Urban<br>2. Rural | |
| 1.4. Kebele (write in full names) | …………………….. | |
| 1.5. Age (in full years) | ………………………. | |
| 1.6. Ethnicity (Circle where appropriate)<br>Tigray (Circle where appropriate) | 1. Amhara<br>2. Afar<br>Other, please specify______________ | |
| 1.7. Sex | 1. Male<br>2. Female | |
| 1.8. Religion | 1. Orthodox<br>2. Muslim<br>Other, please specify_________________________ | |
| 1.9. Marital status | Single/underage<br>Married<br>Divorced<br>Widowed | |
| 1.10. Occupation | Student/underage<br>Farmer<br>Employed<br>Housewife<br>Merchant<br>Daily Laborer<br>Job seeker | |
| 1.11. Educational status | 1. Illiterate<br>2. 1-8<br>3. 9-2<br>4. College and above | |
| **2. Behavioral Data** | **Responses** | **remark** |
| 2.1. Do you currently chew khat? (Circle where appropriate) | 1. Yes<br>2. No | |
| 2.2. How frequent do you chew khat? (Circle where appropriate) | 1. Daily<br>2. Weekly (Once a week)<br>3. Monthly (1-2 times a month)<br>4. Occasional | |
| 2.3. Did use to chew khat in the past? (Circle where appropriate) | 1. Yes<br>2. No | |

| | | |
|---|---|---|
| 2.4. For how long (how many years) have you been chewing khat since you started? | …………………………………… | |
| 6. Do you currently drink alcohol? (Circle where appropriate) | 1. Yes<br>2. No | |
| 7. What type of alcohol do you drink? (Circle where appropriate) | 1. Tela<br>2. Beer<br>3. Areki/Katikala<br>4. Gin<br>5. Burki<br>6. Whiskey<br>7. Mix of alcohol<br>other local beverages, please specify | |
| 2.9. How much alcohol do you drink at once (one episode) (please specify the amount, and measurement in front of each beverage) | 1. Tela, amount_____(measurement_______)<br>2. Beer, amount______(measurement______)<br>3. Areki/Katikala (amount____(measurement ____)<br>4. Gin amount______(measurement _______)<br>5. Bukri amount______(measurement_______)<br>6. Whiskey, mount______(measurement________)<br>7. Mix of alcohol, amount_____(measurement_________) | |
| How frequent do you drink alcohol? | 1. Once in a month<br>2. < three times a week<br>3. times- daily a week | |
| Have you ever been diagnosed with diabetes mellitus (Type 1, Type 2, or gestational diabetes)?" | 1. Yes<br>2. No | |
