# Supplementary material for: Biomarkers of chronic liver disease and their determinants in northern Ethiopia: Evaluating the synergistic impact of HBV and Schistosoma mansoni and the contribution of metabolic and lifestyle factors to liver injury
Source: PLoS One. 2026 Jun 22;21(6):e0352266. doi: 10.1371/journal.pone.0352266 (PMC13286152; doi:10.1371/journal.pone.0352266)
Supplement: S3_File — (PDF) [file pone.0352266.s003.pdf]

Laboratory Results Reporting format

Hepatitis B survey using HBsAg rapid test and *S.mansoni* survey using Kato-Katz Technique

S.NO	Participant's Code	S. mansoni result		HBsAg result		Date of specimen examination (dd/mm/yy)	Remark
		Positive	Negative	Positive	Negative		

***Examined by: Name:* ____________________________ *Signature:* _________ *Date:* ________**
