# Supplementary material for: Biomarkers of chronic liver disease and their determinants in northern Ethiopia: Evaluating the synergistic impact of HBV and Schistosoma mansoni and the contribution of metabolic and lifestyle factors to liver injury
Source: PLoS One. 2026 Jun 22;21(6):e0352266. doi: 10.1371/journal.pone.0352266 (PMC13286152; doi:10.1371/journal.pone.0352266)
Supplement: S4_File — (PDF) [file pone.0352266.s004.pdf]

Reporting format for Biomarkers of Liver function

Test	Test result	Reference range	Remark
AST(SGOT)		Male: 5- 40 U/L<br>Female:5-32 U/L	
ALT(SGPT)		Male 5-41 U/L<br>Females 5-- 33 U/L	
ALP		Male 40-129 U/L<br>Female 35-104 U/L	
Direct billirubin		≤ 0.30 g/dL	
Total billirubin		<1.2 g/dL	
Albumin		3.97-4.94 g/dL	
Total protein		6.6-8.7 g/dL	
Date:______________________<br>signature:__________________			
