# Supplementary material for: Biomarkers of chronic liver disease and their determinants in northern Ethiopia: Evaluating the synergistic impact of HBV and Schistosoma mansoni and the contribution of metabolic and lifestyle factors to liver injury
Source: PLoS One. 2026 Jun 22;21(6):e0352266. doi: 10.1371/journal.pone.0352266 (PMC13286152; doi:10.1371/journal.pone.0352266)
Supplement: S1_checklist — (DOCX) [file pone.0352266.s005.docx]

Inclusivity in global research

PLOS’ policy on inclusivity in global research aims to improve transparency in the reporting of research performed outside of researchers’ own country or community and ensures that PLOS publications reporting global research adhere to high standards for research ethics and authorship. Authors of relevant research articles may be asked to complete the questionnaire below, which outlines ethical, cultural, and scientific considerations specific to inclusivity in global research. This questionnaire may be requested when researchers have travelled to a different country to conduct research, if research uses samples collected in another country, research with Indigenous populations or their lands, or if research is on cultural artefacts. Researchers travelling to another country solely to use laboratory equipment will not normally be required to complete the questionnaire. However, the questionnaire can be requested at the journal’s discretion for any submission – if you have been requested to complete this questionnaire by the PLOS journal you submitted to, please do so.

Please complete the questionnaire below and include this as a Supporting Information file with your manuscript. Note that if your paper is accepted for publication, this checklist will be published with your article in the supporting information files. Please ensure that you reference the checklist in the main body of your manuscript. We suggest adding a subsection ‘Inclusivity in global research’ to your Methods section and adding the following sentence: “Additional information regarding the ethical, cultural, and scientific considerations specific to inclusivity in global research is included in the Supporting Information (SX Checklist)”

The questions have been designed to be applicable to a wide range of study types, and there are subsections for both human subjects research and non-human subjects research. If any of the questions are not relevant to your research please mark them as “N/A” as appropriate.

**Ethical considerations, permits and authorship**

*This section is applicable to all research types.*

Provide details as to who granted permissions and/or consent for the study to take place in the Methods section of your manuscript. This should include the names of **all** ethics boards, governmental organizations, community leaders or other bodies that provided approval for the study. If individuals provided approval refer to these people by their role or title but do not list their name(s).

Reported on page number 8 (Instutional review board of Aklilu Lemma Instutue of Pathobiolgy, Addis Ababa University)

If there were any deviations from the study protocol after approval was obtained please provide details of these changes in the Methods section of your manuscript.
Did this study involve local collaborators that are residents of the country where the research was conducted or members of the community studied? If you do not have any authors from said communities, please provide an explanation for this below.

Reported on page number: N/A

Yes. This study was led and conducted by a research team that is primarily based in Ethiopia. The corresponding author (Gessessew Bugssa) and several co-authors (Dr. Nega Berhe, Dr. Girmay Medhin, and Professor Tilahun Teklehaymanot) are residents of Ethiopia and faculty members at Addis Ababa University and Mekelle University. Furthermore, the study was conducted within the Tigray Region, where the local community was directly involved in the data collection process, and the laboratory analyses were performed at Mekelle University College of Health Sciences.

Everyone listed as an author should meet PLOS’ criteria for authorship and all individuals who meet these criteria should be included in the author byline, rather than the acknowledgements. For further information please see the journal’s Authorship Policy.

**Human subjects research (e.g. health research, medical research, cross-cultural psychology)**

Did you obtain written informed consent from a representative of the local community or region before the research took place? How did you establish who speaks for the community? Details of written informed consent obtained from study participants should be reported separately in the Methods section of your manuscript.

In our study context, community representation was established through a two-tiered approach. First, we secured formal written permission from the Tigray Regional Health Bureau and the Alamata Woreda Health Office, which are the legally recognized administrative authorities for health research in the region.

Second, we engaged directly with Health Extension Workers (HEWs) and their supervisors. As these individuals live and work within the community, they served as the primary communication actors and bridged the research team and the local population. Their involvement ensured that the study was introduced in a culturally appropriate manner, respecting local norms and fostering community trust.

Individual consent, assent, and parental permission were subsequently obtained from participants as detailed in our Methods section.

How did members of the local community provide input on the aims of the research investigation, its methodology, and its anticipated outcome(s)?

Community input was facilitated through established local health structures and consultative meetings with key community actors. This process ensured the research was culturally sensitive and aligned with local health priorities.

Aims and Outcomes: Before data collection, we held consultative meetings with Health Extension workers and their supervisors, and Woreda Health Office officials. During these sessions, the aim of the research was presented. Accordingly, they provided feedback on the study's relevance to local health challenges and helped refine outcomes to ensure the data would be actionable for regional and district level health planning.

Methodological input: The Health Extension Workers (HEWs), who are permanent residents within the community, provided critical input and specifically they advised on the most appropriate times and locations for conducting interviews and sample collection to minimize disruption to the participants' daily and agricultural activities. Besides, they also advised not to conduct the interview and sample collection during market days. They also reviewed the communication approach to ensure the study’s purpose was explained in culturally appropriate language.

Anticipated Outcome: In response to community interest, we established a plan to share finding summaries with the Woreda Health Offices to help improve local health education and screening programs.

When engaging with the local community, how did you ensure that the informed consent documents and other materials could be understood by local stakeholders?

To ensure that informed consent documents and study materials were fully understood by all local stakeholders, we implemented the following measures:

- **Language Translation:** All consent forms, assent documents, and questionnaires were translated from English into **Tigrigna and Amharic**, the local languages of the community. These translations were then translated back to English by an independent expert to ensure technical accuracy and cultural relevance.
- **Used simple language:** We avoided complex medical jargon, instead using descriptive terms that align with local understandings of health and illness.
- **Engagement of Health Extension Workers (HEW):** HEWs who are native speakers and trusted community members provided oral explanations of the documents. This allowed participants to ask questions in their primary language before signing or providing a thumbprint.

Will the findings of the research be made available in an understandable format to stakeholders in the community where the study was conducted (e.g. via a presentation, summary report, copies of publications, etc.)? Please provide details of how this will be achieved.

Yes, we have established a plan to share the research findings with the local community in an accessible format. We will provide summary reports translated into Tigrigna and/or Amharic to the Tigray Regional Health Bureau and Alamata Woreda Health Office to assist in regional health planning. Additionally, we will conduct briefing sessions with Woreda(local) Health Authorities to integrate key insights into their community health education activities. As this study will be published in PLOS ONE, an Open Access journal, the full manuscript will be freely available online, and copies will be shared with Mekelle University and local health authorities to ensure long-term local access.

**Non-human subjects research using specimens/ animals collected as part of the study, or those housed in archival collections. Examples include archaeology, paleontology, botany and zoology.**

Did the permission you obtained from a local authority to perform the study include an agreement on access to outputs and benefit sharing? This may include procedures to enable fair distribution of the benefits and resources arising from the research performed. Please include any details of Prior Informed Consent and Benefit Sharing Agreements obtained. These may be required by field-specific regulations, for example the Convention on Biological Diversity (CBD) and the associated Nagoya Protocol.

As part of our informed consent, we committed to sharing de-identified data summaries and key findings with these authorities to directly inform local health policy and clinical guidelines. The research benefits the community through the provision of diagnostic feedback to local health facilities and the training of Health Extension Workers on liver disease prevention and management. This ensures a fair distribution of knowledge and resources to improve patient care within the community, adhering to regional ethical standards and public health research regulations.

If the material used in your study was imported, please A) provide the year it was imported and B) indicate whether permits were obtained to import/export the materials used, C) provide details of any permits obtained. If this information is not available, please indicate this.

N/A

If you used archival specimens, please state how the material used in your study was acquired by the institute it is held in and provide details of any permits obtained for the original excavations/ sample collection. If this information is not available, please indicate this.

N/A

How was the potential cultural significance of the materials collected in your study to local communities considered in your research design? Were Indigenous peoples and/or local researchers and institutions involved with archaeological excavations / collection of specimens? If so, please provide a description of their involvement.

N/A

If your manuscript includes photographs of human remains please indicate whether authors obtained permission from descendants or affiliated cultural communities to do so.

N/A
