# Supplementary material for: Biomarkers of chronic liver disease and their determinants in northern Ethiopia: Evaluating the synergistic impact of HBV and Schistosoma mansoni and the contribution of metabolic and lifestyle factors to liver injury
Source: PLoS One. 2026 Jun 22;21(6):e0352266. doi: 10.1371/journal.pone.0352266 (PMC13286152; doi:10.1371/journal.pone.0352266)
Supplement: Attachment — Submitted filename: Response to Reviewers.docx [file pone.0352266.s006.docx]

**Point-by-point response to reviewers’ comments**

**Date: 15^th^ May, 2026.**

**To: Editorial Board, PlosOne**

**Manuscript ID**: PONE-D-26-09027

**Dear Dr. Seth Agyei Domfeh**,

Thank you for the opportunity to submit a revised version of our manuscript. The feedback provided by the reviewers was indeed valid and helped us significantly improve the clarity and depth of our work. In response, each e comment given by the reviewers has been carefully considered and incorporated into the revised manuscript. We have made the necessary amendments to ensure that the study accurately reflects the key findings and objectives of the research.

We have implemented the following key updates to the manuscript;

**Comprehensive Response to Reviewers’ Comments**: As a primary component of this revision, we have addressed each reviewer's query with specific clarifications and supporting data. These updates are reflected in the unmarked manuscript and are highlighted in the “Track Changes” version to ensure full transparency regarding the amendments.

**Title Modification**: We have refined the title to better align with the study’s primary focus, ensuring it accurately reflects our investigation into both independent determinants and specific interactions.

**Statistical Analysis**: To provide a deeper understanding of how different variables interact, we have employed multivariable hierarchical logistic regression. This approach allows for a clearer distinction between infectious, metabolic, and lifestyle determinants.

**Data Visualization**: The results of this hierarchical analysis and the interactions between factors are now illustrated using a forest plot (Figure 2).

**New tables**: Three new tables have been added to provide a more detailed breakdown of the findings namely: Table 4, a cross-tabulation table showing the distribution of elevated ALT across various socio-demographic, infectious, lifestyle, and metabolic risk factors; Table 5, a table detailing the distribution of AST/ALT ratios among the investigated risk factors; and Table 6 about model summary for hierarchical logistic regression

**Additional References**: In response to requests for further elaboration, we have incorporated seven additional references to provide a more comprehensive context. These citations are included in the unmarked manuscript and are also highlighted in the “Track Changes” version.

Moreover, we would like to acknowledge the concerns raised by the PlosOne editor regarding the Data Privacy and Ethics. Accordingly, we have removed all potentially identifiable participant data to ensure confidentiality. Furthermore, we have not included the raw interview transcripts in the supporting information to ensure confidentiality. The data provided in the revised supporting information consists only of the de-identified data and blank research questionnaire and laboratory reporting formats used in the study. To this end, the study was conducted under full ethical clearance from Institutional review board of (IRB) of Aklilu Lemma Institute of Pathobiology, Addis Ababa University. We confirm that formal informed consent, participant assent, and parental permission were obtained for all individuals prior to the interviewing process, with strict ethical standards.

**Below are our point-by-point responses to the reviewers’ comments.**

**Reviewer 1:**

1. **Reviewer’s comment:** *A substantial proportion of the study population belongs to the 5 to 14 year age group (27.8%), but some behavioral variables such as alcohol consumption and khat chewing are analyzed across the entire cohort. It is unclear whether these behavioral variables were assessed only among adults or among all participants. If minors were included in these analyses, this raises ethical and methodological concerns. Age-adjusted analyses or age-restricted behavioral analyses (e.g., ≥15 years) may be necessary.*

**Authors’ Response**: We sincerely thank the reviewer for this insightful observation. We acknowledge that behavioral risk factors like alcohol consumption and khat chewing have distinct ethical and exposure profiles in the pediatric population compared to adults, where a high proportion of non-exposed minors could dilute their observed effects. Following international standards like WHO’s 10 year age intervals (to maintain statistical power in large datasets), we categorized groups as 5-14 (pediatric), 15-24 (youth), etc. To address this, we used a logic-based coding approach in the multivariable logistic regression: participants aged 5-14 were coded as '0' (non-exposed) for alcohol and khat, and this group served as the reference category for age as it was also suggested by the reviewer. This effectively partitioned variance (age related differences), isolating the independent effects of these behaviors in eligible older groups while adjusting for other risks (e.g., HBV, *S.mansoni*, DM) across the full cohort (N=767).

**Revisions in the manuscript**: We have updated the Results section (Fig. 2), multivariable model descriptions (lines 317-340) to reflect these refined analyses.

2. **Reviewer’s comment**: ……. whether age-adjusted models were used in the regression analysis.

**Authors’ Response**: Regarding the regression analysis, we utilized age-adjusted multivariable logistic regression models. Because age was forced into the final multivariable model as an a priori confounder, the reported Adjusted Odds Ratios (AOR) for alcohol and khat use are mathematically calculated while holding the age variable constant.

3. **Reviewer’s Comment**: *The study defines abnormal liver biomarkers using fixed thresholds for ALT and ALP. However, these appear to be adult reference ranges, despite inclusion of children. This is particularly problematic for ALP, which is physiologically elevated in children due to bone growth. Authors should clarify whether Age-specific laboratory reference ranges were applied, or adult reference ranges were used for all participants. Failure to address this could bias estimates of abnormal biomarkers.*

**Authors’ Response**: We thank the reviewer for pointing out this. We fully agree that using adult reference ranges for pediatric populations, particularly for ALP, would lead to a significant overestimation of biochemical abnormality. During the data collection and initial analysis, age-specific reference intervals were indeed applied at the laboratory level. For ALP, thresholds were adjusted according to pediatric norms to account for active bone growth (lines 167-169).

**Revision in the manuscript**: The normal ranges are incorporated in the methods section, laboratory Analysis sub-section (lines 167-169). Besides, the findings are interpreted cautiously in the discussion section (lines 371-381).

4. **Reviewer’s comment**: *The study focuses heavily on abnormal ALT as an indicator of liver disease. However, ALT elevation does not necessarily indicate chronic liver disease and may reflect transient liver injury. Possible causes include Viral hepatitis, Parasitic infection, Metabolic dysfunction, Drug or toxin exposure. Therefore, statements suggesting a ‘huge burden of undiagnosed liver disease’ should be interpreted cautiously.*

**Authors’ Response**: We sincerely appreciate the reviewer’s caution regarding the interpretation of ALT. We agree that a single elevated ALT measurement can reflect transient liver injury (acute) rather than established chronic liver disease (CLD).

**Revision in the manuscript**: This has been addressed in the discussion section (lines 361-362).

5. **Reviewer’s comment***: The manuscript reports higher ALT levels among participants aged 5 to 14 years, which is unusual and requires deeper exploration.*

**Authors’ Response:** We appreciate the reviewer for noting this atypical finding. We acknowledge that our finding of higher ALT levels in the 5-14 years age group deviates from the usual trends. In large-scale population studies in non-endemic regions, ALT levels typically show a positive correlation with age, largely driven by the accumulation of metabolic risk factors in adulthood as opposed to pediatric reference ranges who have significantly lower ranges than the adult at base line. However, we would like to justify that in the context of Northern Ethiopia, this reverse trend is driven by unique epidemiological landscape of the area where there is early-life burden of Schistosomiasis and immune-active *Hepatitis B infections* in the pediatric population which can cause elevated ALT/or high viral loads. This explains why our community findings differ from the trends seen or metabolic driven studies.

**Revisions in the manuscript**: We have revised the discussion to address these discrepancies, as detailed in lines 381-387.

6. **Reviewer’s comment***: The description of the regression analysis lacks several important details. Although bivariate logistic regression followed by multivariable analysis was conducted, whether key confounders (age and sex) were considered for the adjustments.*

**Authors’ Response**: We thank the reviewer for this important request for clarification. We agree that adjusting for fundamental demographic confounders is essential for the validity of the multivariable model. In our analysis, we followed a robust two-step approach to confounding. Initially, potential risk factors with a p-value <0.25 in the bivariate analysis were considered for multivariable analysis. Furthermore, we treated age and sex as a priori confounders to be forced into the final model regardless of their initial significance.

**Revisions in the manuscript**: We have updated the methods section under Data Analysis section (202-205) and the results section (lines 317-340) to explicitly state that age and sex were included in the multivariable model as fixed covariates to control for potential confounding.

7. **Reviewer’s comment**: *The authors used bivariate logistic regression models with P < 0.25 in the bivariate analysis. Why was p value <0.25 selected? If p< 0.05 is considered, the conclusion will be different?*
   **Authors’ Response**: We appreciate the reviewer’s question regarding our selection of the p < 0.25 threshold for multivariable entry. The choice of p < 0.25 is a recognized strategy in purposeful selection of covariates, as recommended by Bursac et.al (2008) and Hosmer and Lemeshow (2000). Using a strict P<.05 cut-off in the bivariate analysis might fails to identify variables that are known to be important confounders or those that become significant only when adjusted for other covariates. By using a broader screening threshold (P<.25), we ensure that we do not prematurely exclude variables that contribute to a more stable and biologically plausible multivariable model.
8. **Reviewer’s comment**: *The reported association between HBV infection and abnormal ALT (aOR ≈ 18) is extremely strong and requires careful interpretation.*
   **Author’s Response**: We thank the reviewer for highlighting this. Although the value of aOR has change a from 18 to 13 (after adjusting for age), we agree that such a large effect size warrants cautious interpretation and we have addressed it thoroughly in our revisions. The high association between HBV and elevated ALT underscores the potency of viral hepatitis as a driver of liver injury in this community. This magnitude is consistent with the natural history of HBV in endemic regions, where early-life infection often leads to prolonged immune-active phases characterized by intense hepatocellular damage and high transaminase levels. While the odds ratio is high, it highlights the urgent need for expanded screening and antiviral intervention to mitigate this profound risk.

**Revisions in the manuscript:** We have expanded the discussion (lines 395-399) to contextualize this finding against regional HBV epidemiology.

**Minor comments (Reviewer 1)**

1. The text indicates that most participants were from rural areas, while the table shows 67.5% of urban residence. This inconsistency must be corrected.

**Response**: We thank the reviewer for pointing out this discrepancy. There was a labeling error in the table where the ‘rural’ and ‘urban’ categories were misplaced. This has now been corrected in the revised table to ensure consistency with the text.

2. Albumin is reported as mg/dL, whereas the correct unit is g/dL. This error should be corrected throughout the manuscript.

**Response**: Thank you for the observation. The units for albumin have been corrected to g/dL throughout the revised manuscript to ensure clinical and scientific accuracy (revisions are shown).

3. Several minor errors are present. For examples, “Sever” → Severe, “Mini.” → Min, “73,5” → 73.5.

**Response**: We thank the reviewer for the careful proofreading. We have corrected the identified errors and have made a thorough review of the entire manuscript to correct any typographical errors.

**Reviewer 2:**

1. **Reviewer’s comment**: *The major concern in the study is the single-time measurements of liver function biomarkers. Although the study covers a 5-year period liver function was assessed only at one time point this may capture transient abnormalities rather than chronic liver disease, and lead to misclassification of the liver health status of participants.*

**Authors’ Response**: We sincerely thank the reviewer for identifying this discrepancy regarding the study period. We apologize for the confusion caused by the dates provided in the original abstract. To clarify, this research was conducted as part of a PhD study. While the entire project-including laboratory analysis, data entry, statistical processing, and write-up was extended from 2019 to 2024, the actual field data collection and specimen sampling occurred between December 2019 and June 2020. The study was intended to be a community-based cross-sectional study. We also agree that a single-point measurement reflects a snapshot of the population. We have now corrected the dates in the Abstract (line 33) and Methods section (120) to accurately reflect the 6 months data collection period (December 2019-June 2020) to ensure readers understand the cross-sectional nature of the data. It was also indicated as a limitation in the discussion section acknowledging that single-time measurements may capture transient elevations in biomarkers considering the cross-sectional nature of the study.

**Revisions in the manuscript**: revisions made in the abstract section line 33-34.

2. **Reviewer’s Comment**: *Interpretation of liver health is based on analysis of each biomarker independently (AST/ALT ratios not computed).*
   **Author’s Response**: We would like to sincerely thank the reviewer for highlighting the importance of these parameters. In our initial analysis and earlier drafts, we had calculated the AST/ALT ratio (De Ritis ratio). However, we chose to omit these results from the previous version of the manuscript, as we initially felt they might serve as weaker independent indicators of liver injury compared to absolute biomarker values.

Upon re-evaluating the manuscript in light of your comments, we agree that the AST/ALT ratio (AAR) provides critical insights into the pattern of liver injury (distinguishing between alcoholic-hepatocellular with non-alcoholic like patterns) and adds significant value to the clinical interpretation of our findings. We have now incorporated these results into the manuscript. Besides, we have also incorporated additional points on R-value to determine the patterns of liver injury. We are grateful to the reviewer for noting the importance of these parameters, as we believe their inclusion has strengthened the paper.

**Revisions in the manuscript**: We have included the methods section, determination of patterns of liver injury sub-section, we have indicated the AAR ratio (170-172), and the R value on classification of liver injury patterns (line 173-177). In the results section, we have incorporated the AST/ALT ratio (AAR). Besides, the mean AAR, and category were calculated to indicate the etiologies of liver injury as indicated in lines 280-295, and the R-values to determine the patterns of liver injury (lines 296-315). We have also revised the discussion sections based on the AAR and R-value findings (436-455).

3. ***Comment****: Wide age range. The study population varied from 5 to 80 years and raises concerns if mean values can be skewed by the effect of age.*
   **Author Response**: We thank the reviewer for highlighting regarding the broad age range in our study population and agree that liver function biomarkers may differ across life stages. To address this, we conducted age-stratified analyses. Furthermore, in our multivariate logistic regression models, age was included as a confounding variable to ensure that the reported associations for Chronic Liver Disease (CLD) risk factors were independent of participants' age.
4. **Reviewer’s comment**: The study uses liver function biomarkers as indicators of hepatic injury, but these biomarkers cannot determine causality. Hence, it’s important for the authors not to present any identified significance as causality.

**Authors’ Response**: We fully agree with the reviewer’s assessment. Given the cross-sectional nature of the study, we acknowledge that our findings denote statistical associations rather than temporal causality. Accordingly, we have carefully assessed and reviewed the manuscript to eliminate any language implying a cause and effect relationship. We have also explicitly stated this limitation that in the discussion section that this does not indicate causal inference (459-460).

5. **Reviewer’s Comment**: Demographic information are obtained by self-reported data for behavioral factors such as alcohol and khat use. This reliance introduces the potential for reporting bias, as participants may underreport or inaccurately report their behaviors.

**Authors’ Response**: We thank the reviewer for this important observation regarding the nature of self-reported data. We fully acknowledge that relying on participant recall for behavioral factors such as alcohol and khat use introduces the potential for reporting bias, including both recall bias and social desirability bias. To address this, we have explicitly included this point in the limitations section of the manuscript, noting that *‘self-reported behavioral factors, such as alcohol and khat use, might have introduced potential reporting bias’* (lines 464-465*).* To minimize this risk during the study, interviews were conducted in a private and confidential setting by trained data collectors to encourage honest reporting. Furthermore, we used standardized questionnaire to maintain consistency across the study population. We believe that while this remains an inherent limitation of community-based surveys, the reported associations provide a meaningful baseline for understanding liver health determinants in this region.

We hope that these revisions meet the high standards of PLOS ONE and address the concerns raised during the initial review. We remain at your disposal if any further clarification is required.

**Sincerely!**

(The corresponding Author)
