# Supplementary material for: Biomarkers of chronic liver disease and their determinants in northern Ethiopia: Evaluating the synergistic impact of HBV and Schistosoma mansoni and the contribution of metabolic and lifestyle factors to liver injury
Source: PLoS One. 2026 Jun 22;21(6):e0352266. doi: 10.1371/journal.pone.0352266 (PMC13286152; doi:10.1371/journal.pone.0352266)
Supplement: Peer review history

# Peer review: Decision Letter 0

**DOI:** 10.1371/journal.pone.0352266.r001

**Authors:** Seth Domfeh

27 Mar 2026

PONE-D-26-09027

Biomarkers of Chronic Liver Disease and Their Determinants in Northern Ethiopia

PLOS One

Dear Dr. Bugssa,

Thank you for submitting your manuscript to PLOS ONE. After careful consideration, we feel that it has merit but does not fully meet PLOS ONE’s publication criteria as it currently stands. Therefore, we invite you to submit a revised version of the manuscript that addresses the points raised during the review process.

Please submit your revised manuscript by May 11 2026 11:59PM. If you will need more time than this to complete your revisions, please reply to this message or contact the journal office at plosone@plos.org. When you're ready to submit your revision, log on to https://www.editorialmanager.com/pone/ and select the 'Submissions Needing Revision' folder to locate your manuscript file.

Please include the following items when submitting your revised manuscript:

- A letter that responds to each point raised by the academic editor and reviewer(s). You should upload this letter as a separate file labeled 'Response to Reviewers'.
- A marked-up copy of your manuscript that highlights changes made to the original version. You should upload this as a separate file labeled 'Revised Manuscript with Track Changes'.
- An unmarked version of your revised paper without tracked changes. You should upload this as a separate file labeled 'Manuscript'.

If you would like to make changes to your financial disclosure, please include your updated statement in your cover letter. Guidelines for resubmitting your figure files are available below the reviewer comments at the end of this letter.

If applicable, we recommend that you deposit your laboratory protocols in protocols.io to enhance the reproducibility of your results. Protocols.io assigns your protocol its own identifier (DOI) so that it can be cited independently in the future. For instructions see: https://journals.plos.org/plosone/s/submission-guidelines#loc-laboratory-protocols. Additionally, PLOS ONE offers an option for publishing peer-reviewed Lab Protocol articles, which describe protocols hosted on protocols.io. Read more information on sharing protocols at https://plos.org/protocols?utm_medium=editorial-email&utm_source=authorletters&utm_campaign=protocols.

As the corresponding author, your ORCID iD is verified in the submission system and will appear in the published article. PLOS supports the use of ORCID, and we encourage all coauthors to register for an ORCID iD and use it as well. Please encourage your coauthors to verify their ORCID iD within the submission system before final acceptance, as unverified ORCID iDs will not appear in the published article. *Only*   the individual author can complete the verification step; PLOS staff *cannot*   verify ORCID iDs on behalf of authors.

We look forward to receiving your revised manuscript.

Kind regards,

Seth Agyei Domfeh, PhD

Academic Editor

PLOS One

**Journal Requirements:**

1. When submitting your revision, we need you to address these additional requirements.

Please ensure that your manuscript meets PLOS ONE's style requirements, including those for file naming. The PLOS ONE style templates can be found at

https://journals.plos.org/plosone/s/file?id=wjVg/PLOSOne_formatting_sample_main_body.pdf and

https://journals.plos.org/plosone/s/file?id=ba62/PLOSOne_formatting_sample_title_authors_affiliations.pdf

2. Please include a complete copy of PLOS’ questionnaire on inclusivity in global research in your revised manuscript. Our policy for research in this area aims to improve transparency in the reporting of research performed outside of researchers’ own country or community. The policy applies to researchers who have travelled to a different country to conduct research, research with Indigenous populations or their lands, and research on cultural artefacts. The questionnaire can also be requested at the journal’s discretion for any other submissions, even if these conditions are not met.  Please find more information on the policy and a link to download a blank copy of the questionnaire here: https://journals.plos.org/plosone/s/best-practices-in-research-reporting. Please upload a completed version of your questionnaire as Supporting Information when you resubmit your manuscript.

3. Thank you for stating the following in the Acknowledgments Section of your manuscript:

We express our gratitude to Aklilu Lemma Institute of Pathobiology, Addis

Ababa University for providing the opportunity to conduct this research. We also extend our

appreciation of Mekelle University (NORAD Project) for financially supporting the laboratory analysis phase of this study.

We note that you have provided funding information that is not currently declared in your Funding Statement. However, funding information should not appear in the Acknowledgments section or other areas of your manuscript. We will only publish funding information present in the Funding Statement section of the online submission form.

Please remove any funding-related text from the manuscript and let us know how you would like to update your Funding Statement. Currently, your Funding Statement reads as follows:

The author(s) received no specific funding for this work.

Please include your amended statements within your cover letter; we will change the online submission form on your behalf.

4. When completing the data availability statement of the submission form, you indicated that you will make your data available on acceptance. We strongly recommend all authors decide on a data sharing plan before acceptance, as the process can be lengthy and hold up publication timelines. Please note that, though access restrictions are acceptable now, your entire data will need to be made freely accessible if your manuscript is accepted for publication. This policy applies to all data except where public deposition would breach compliance with the protocol approved by your research ethics board. If you are unable to adhere to our open data policy, please kindly revise your statement to explain your reasoning and we will seek the editor's input on an exemption. Please be assured that, once you have provided your new statement, the assessment of your exemption will not hold up the peer review process.

5. We note that there is identifying data in the Supporting Information file < S1 Data file>. Due to the inclusion of these potentially identifying data, we have removed this file from your file inventory. Prior to sharing human research participant data, authors should consult with an ethics committee to ensure data are shared in accordance with participant consent and all applicable local laws.

Data sharing should never compromise participant privacy. It is therefore not appropriate to publicly share personally identifiable data on human research participants. The following are examples of data that should not be shared:

-Name, initials, physical address

-Ages more specific than whole numbers

-Internet protocol (IP) address

-Specific dates (birth dates, death dates, examination dates, etc.)

-Contact information such as phone number or email address

-Location data

-ID numbers that seem specific (long numbers, include initials, titled “Hospital ID”) rather than random (small numbers in numerical order)

Data that are not directly identifying may also be inappropriate to share, as in combination they can become identifying. For example, data collected from a small group of participants, vulnerable populations, or private groups should not be shared if they involve indirect identifiers (such as sex, ethnicity, location, etc.) that may risk the identification of study participants.

Additional guidance on preparing raw data for publication can be found in our Data Policy (https://journals.plos.org/plosone/s/data-availability#loc-human-research-participant-data-and-other-sensitive-data) and in the following article: http://www.bmj.com/content/340/bmj.c181.long.

Please remove or anonymize all personal information (<specific identifying information in file to be removed>), ensure that the data shared are in accordance with participant consent, and re-upload a fully anonymized data set. Please note that spreadsheet columns with personal information must be removed and not hidden as all hidden columns will appear in the published file.

6. We note that this data set consists of interview transcripts. Can you please confirm that all participants gave consent for interview transcript to be published?

If they DID provide consent for these transcripts to be published, please also confirm that the transcripts do not contain any potentially identifying information (or let us know if the participants consented to having their personal details published and made publicly available). We consider the following details to be identifying information:

- Names, nicknames, and initials

- Age more specific than round numbers

- GPS coordinates, physical addresses, IP addresses, email addresses

- Information in small sample sizes (e.g. 40 students from X class in X year at X university)

- Specific dates (e.g. visit dates, interview dates)

- ID numbers

Or, if the participants DID NOT provide consent for these transcripts to be published:

- Provide a de-identified version of the data or excerpts of interview responses

- Provide information regarding how these transcripts can be accessed by researchers who meet the criteria for access to confidential data, including:

a) the grounds for restriction

b) the name of the ethics committee, Institutional Review Board, or third-party organization that is imposing sharing restrictions on the data

c) a non-author, institutional point of contact that is able to field data access queries, in the interest of maintaining long-term data accessibility.

d) Any relevant data set names, URLs, DOIs, etc. that an independent researcher would need in order to request your minimal data set.

For further information on sharing data that contains sensitive participant information, please see: https://journals.plos.org/plosone/s/data-availability#loc-human-research-participant-data-and-other-sensitive-data

If there are ethical, legal, or third-party restrictions upon your dataset, you must provide all of the following details (https://journals.plos.org/plosone/s/data-availability#loc-acceptable-data-access-restrictions):

a) A complete description of the dataset

b) The nature of the restrictions upon the data (ethical, legal, or owned by a third party) and the reasoning behind them

c) The full name of the body imposing the restrictions upon your dataset (ethics committee, institution, data access committee, etc)

d) If the data are owned by a third party, confirmation of whether the authors received any special privileges in accessing the data that other researchers would not have

e) Direct, non-author contact information (preferably email) for the body imposing the restrictions upon the data, to which data access requests can be sent

7. If the reviewer comments include a recommendation to cite specific previously published works, please review and evaluate these publications to determine whether they are relevant and should be cited. There is no requirement to cite these works unless the editor has indicated otherwise.

8. Please review your reference list to ensure that it is complete and correct. If you have cited papers that have been retracted, please include the rationale for doing so in the manuscript text, or remove these references and replace them with relevant current references. Any changes to the reference list should be mentioned in the rebuttal letter that accompanies your revised manuscript. If you need to cite a retracted article, indicate the article’s retracted status in the References list and also include a citation and full reference for the retraction notice.

[Note: HTML markup is below. Please do not edit.]

Reviewers' comments:

Reviewer's Responses to Questions

**Comments to the Author**

1. Is the manuscript technically sound, and do the data support the conclusions?

The manuscript must describe a technically sound piece of scientific research with data that supports the conclusions. Experiments must have been conducted rigorously, with appropriate controls, replication, and sample sizes. The conclusions must be drawn appropriately based on the data presented.

Reviewer #1: Partly

Reviewer #2: Yes

**********

2. Has the statistical analysis been performed appropriately and rigorously?

Reviewer #1: No

Reviewer #2: Yes

**********

3. Have the authors made all data underlying the findings in their manuscript fully available?

The PLOS Data policy requires authors to make all data underlying the findings described in their manuscript fully available without restriction, with rare exception (please refer to the Data Availability Statement in the manuscript PDF file). The data should be provided as part of the manuscript or its supporting information, or deposited to a public repository. For example, in addition to summary statistics, the data points behind means, medians and variance measures should be available. If there are restrictions on publicly sharing data—e.g. participant privacy or use of data from a third party—those must be specified.

Reviewer #1: Yes

Reviewer #2: Yes

**********

4. Is the manuscript presented in an intelligible fashion and written in standard English?

PLOS ONE does not copyedit accepted manuscripts, so the language in submitted articles must be clear, correct, and unambiguous. Any typographical or grammatical errors should be corrected at revision, so please note any specific errors here.

Reviewer #1: Yes

Reviewer #2: Yes

**********

5. Review Comments to the Author

Please use the space provided to explain your answers to the questions above. You may also include additional comments for the author, including concerns about dual publication, research ethics, or publication ethics. (Please upload your review as an attachment if it exceeds 20,000 characters)

**Reviewer #1:**   This manuscript investigates the prevalence and determinants of liver function biomarker abnormalities in a community-based population. The study addresses an important public health issue in a region where infectious diseases such as Hepatitis B and Schistosomiasis remain endemic and where lifestyle factors such as Catha edulis consumption may influence liver health.

The community-based design and relatively large sample size (n = 767) are strengths. However, several methodological, statistical, and interpretational issues require clarification or revision before the manuscript can be considered for publication.

Major:

1. A substantial proportion of the study population belongs to the 5 to 14 year age group (27.8%), but some behavioral variables such as alcohol consumption and khat chewing are analyzed across the entire cohort. It is unclear whether these behavioral variables were assessed only among adults or among all participants. If minors were included in these analyses, this raises ethical and methodological concerns. Age-adjusted analyses or age-restricted behavioral analyses (e.g., ≥15 years) may be necessary.

2. Whether age-adjusted models were used in the regression analysis.

3. The study defines abnormal liver biomarkers using fixed thresholds for ALT and ALP. However, these appear to be adult reference ranges, despite inclusion of children. This is particularly problematic for ALP, which is physiologically elevated in children due to bone growth. Authors should clarify whether Age-specific laboratory reference ranges were applied, or adult reference ranges were used for all participants. Failure to address this could prevalence bias estimates of abnormal biomarkers.

4. The study focuses heavily on abnormal ALT as an indicator of liver disease. However, ALT elevation does not necessarily indicate chronic liver disease and may reflect transient liver injury. Possible causes include Viral hepatitis, Parasitic infection, Metabolic dysfunction, Drug or toxin exposure. Therefore, statements suggesting a ‘huge burden of undiagnosed liver disease’ should be interpreted cautiously.

5. The manuscript reports higher ALT levels among participants aged 5 to 14 years, which is unusual and requires deeper exploration.

6. The description of the regression analysis lacks several important details. Although bivariate logistic regression followed by multivariable analysis was conducted, whether key confounders (age and sex) were considered for the adjustments.

7. The authors used bivariate logistic regression models with P < 0.25 in the bivariate analysis. Why was p value <0.25 selected? If p< 0.05 is considered, the conclusion will be different?

8. The reported association between HBV infection and abnormal ALT (AOR ≈ 18) is extremely strong and requires careful interpretation.

Minor:

1. The text indicates that most participants were from rural areas, while the table shows 67.5% of urban residence. This inconsistency must be corrected.

2. Albumin is reported as mg/dL, whereas the correct unit is g/dL. This error should be corrected throughout the manuscript.

3. Several minor errors are present. For examples, “Sever” → Severe, “Mini.” → Min, “73,5” → 73.5

**Reviewer #2:**   In the article ‘Biomarkers of Chronic Liver Disease and Their Determinants in Northern Ethiopia’ Gessessew et al examined the prevalence and determinants of biomarkers indicative of chronic liver disease in a community in Northern Ethiopia. The authors conducted a cross-sectional study involving 767 participants, assessing liver function biomarkers and infections. The authors found that nearly a quarter of participants had abnormal liver biomarkers, with significant associations between elevated ALT and factors such as HBV infection, S. mansoni infection, khat chewing, and diabetes mellitus.

Major concern:

1. The major concern in the study is the single-time measurements of liver function biomarkers. Although the study covers a 5-year period liver function was assessed only at one time point this may capture transient abnormalities rather than chronic liver disease, and lead to misclassification of the liver health status of participants.

2. Interpretation of liver health is based on analysis of each biomarker independently (AST/ALT ratios not computed).

3. Wide age range. The study population varied from 5 to 80 years and raises concerns if mean values can be skewed by the effect of age.

4. The study uses liver function biomarkers as indicators of hepatic injury, but these biomarkers cannot determine causality. Hence it’s important for the authors not to present any identified significance as causality.

5. Demographic information are obtained by self-reported data for behavioural factors such as alcohol and khat use. This reliance introduces the potential for reporting bias, as participants may underreport or inaccurately report their behaviours.

**********

6. PLOS authors have the option to publish the peer review history of their article (what does this mean?). If published, this will include your full peer review and any attached files.

If you choose “no”, your identity will remain anonymous but your review may still be made public.

**Do you want your identity to be public for this peer review?**   For information about this choice, including consent withdrawal, please see our Privacy Policy.

Reviewer #1: No

Reviewer #2: No

**********

[NOTE: If reviewer comments were submitted as an attachment file, they will be attached to this email and accessible via the submission site. Please log into your account, locate the manuscript record, and check for the action link "View Attachments". If this link does not appear, there are no attachment files.]

To ensure your figures meet our technical requirements, please review our figure guidelines: https://journals.plos.org/plosone/s/figures

You may also use PLOS’s free figure tool, NAAS, to help you prepare publication quality figures: https://journals.plos.org/plosone/s/figures#loc-tools-for-figure-preparation.

NAAS will assess whether your figures meet our technical requirements by comparing each figure against our figure specifications.

# Author comment: Author response to Decision Letter 1

**DOI:** 10.1371/journal.pone.0352266.r002

15 May 2026

Point-by-point response to reviewers’ comments

Date: 15th May, 2026.

To: Editorial Board, PlosOne

Manuscript ID: PONE-D-26-09027

Dear Dr. Seth Agyei Domfeh,

Thank you for the opportunity to submit a revised version of our manuscript. The feedback provided by the reviewers was indeed valid and helped us significantly improve the clarity and depth of our work. In response, each e comment given by the reviewers has been carefully considered and incorporated into the revised manuscript. We have made the necessary amendments to ensure that the study accurately reflects the key findings and objectives of the research.

We have implemented the following key updates to the manuscript;

Comprehensive Response to Reviewers’ Comments: As a primary component of this revision, we have addressed each reviewer's query with specific clarifications and supporting data. These updates are reflected in the unmarked manuscript and are highlighted in the “Track Changes” version to ensure full transparency regarding the amendments.

Title Modification: We have refined the title to better align with the study’s primary focus, ensuring it accurately reflects our investigation into both independent determinants and specific interactions.

Statistical Analysis: To provide a deeper understanding of how different variables interact, we have employed multivariable hierarchical logistic regression. This approach allows for a clearer distinction between infectious, metabolic, and lifestyle determinants.

Data Visualization: The results of this hierarchical analysis and the interactions between factors are now illustrated using a forest plot (Figure 2).

New tables: Three new tables have been added to provide a more detailed breakdown of the findings namely: Table 4, a cross-tabulation table showing the distribution of elevated ALT across various socio-demographic, infectious, lifestyle, and metabolic risk factors; Table 5, a table detailing the distribution of AST/ALT ratios among the investigated risk factors; and Table 6 about model summary for hierarchical logistic regression

Additional References: In response to requests for further elaboration, we have incorporated seven additional references to provide a more comprehensive context. These citations are included in the unmarked manuscript and are also highlighted in the “Track Changes” version.

Moreover, we would like to acknowledge the concerns raised by the PlosOne editor regarding the Data Privacy and Ethics. Accordingly, we have removed all potentially identifiable participant data to ensure confidentiality. Furthermore, we have not included the raw interview transcripts in the supporting information to ensure confidentiality. The data provided in the revised supporting information consists only of the de-identified data and blank research questionnaire and laboratory reporting formats used in the study. To this end, the study was conducted under full ethical clearance from Institutional review board of (IRB) of Aklilu Lemma Institute of Pathobiology, Addis Ababa University. We confirm that formal informed consent, participant assent, and parental permission were obtained for all individuals prior to the interviewing process, with strict ethical standards.

Below are our point-by-point responses to the reviewers’ comments.

Reviewer 1:

1. Reviewer’s comment: A substantial proportion of the study population belongs to the 5 to 14 year age group (27.8%), but some behavioral variables such as alcohol consumption and khat chewing are analyzed across the entire cohort. It is unclear whether these behavioral variables were assessed only among adults or among all participants. If minors were included in these analyses, this raises ethical and methodological concerns. Age-adjusted analyses or age-restricted behavioral analyses (e.g., ≥15 years) may be necessary.

Authors’ Response: We sincerely thank the reviewer for this insightful observation. We acknowledge that behavioral risk factors like alcohol consumption and khat chewing have distinct ethical and exposure profiles in the pediatric population compared to adults, where a high proportion of non-exposed minors could dilute their observed effects. Following international standards like WHO’s 10 year age intervals (to maintain statistical power in large datasets), we categorized groups as 5-14 (pediatric), 15-24 (youth), etc. To address this, we used a logic-based coding approach in the multivariable logistic regression: participants aged 5-14 were coded as '0' (non-exposed) for alcohol and khat, and this group served as the reference category for age as it was also suggested by the reviewer. This effectively partitioned variance (age related differences), isolating the independent effects of these behaviors in eligible older groups while adjusting for other risks (e.g., HBV, S.mansoni, DM) across the full cohort (N=767).

Revisions in the manuscript: We have updated the Results section (Fig. 2), multivariable model descriptions (lines 317-340) to reflect these refined analyses.

2. Reviewer’s comment: ……. whether age-adjusted models were used in the regression analysis.

Authors’ Response: Regarding the regression analysis, we utilized age-adjusted multivariable logistic regression models. Because age was forced into the final multivariable model as an a priori confounder, the reported Adjusted Odds Ratios (AOR) for alcohol and khat use are mathematically calculated while holding the age variable constant.

3. Reviewer’s Comment: The study defines abnormal liver biomarkers using fixed thresholds for ALT and ALP. However, these appear to be adult reference ranges, despite inclusion of children. This is particularly problematic for ALP, which is physiologically elevated in children due to bone growth. Authors should clarify whether Age-specific laboratory reference ranges were applied, or adult reference ranges were used for all participants. Failure to address this could bias estimates of abnormal biomarkers.

Authors’ Response: We thank the reviewer for pointing out this. We fully agree that using adult reference ranges for pediatric populations, particularly for ALP, would lead to a significant overestimation of biochemical abnormality. During the data collection and initial analysis, age-specific reference intervals were indeed applied at the laboratory level. For ALP, thresholds were adjusted according to pediatric norms to account for active bone growth (lines 167-169).

Revision in the manuscript: The normal ranges are incorporated in the methods section, laboratory Analysis sub-section (lines 167-169). Besides, the findings are interpreted cautiously in the discussion section (lines 371-381).

4. Reviewer’s comment: The study focuses heavily on abnormal ALT as an indicator of liver disease. However, ALT elevation does not necessarily indicate chronic liver disease and may reflect transient liver injury. Possible causes include Viral hepatitis, Parasitic infection, Metabolic dysfunction, Drug or toxin exposure. Therefore, statements suggesting a ‘huge burden of undiagnosed liver disease’ should be interpreted cautiously.

Authors’ Response: We sincerely appreciate the reviewer’s caution regarding the interpretation of ALT. We agree that a single elevated ALT measurement can reflect transient liver injury (acute) rather than established chronic liver disease (CLD).

Revision in the manuscript: This has been addressed in the discussion section (lines 361-362).

5. Reviewer’s comment: The manuscript reports higher ALT levels among participants aged 5 to 14 years, which is unusual and requires deeper exploration.

Authors’ Response: We appreciate the reviewer for noting this atypical finding. We acknowledge that our finding of higher ALT levels in the 5-14 years age group deviates from the usual trends. In large-scale population studies in non-endemic regions, ALT levels typically show a positive correlation with age, largely driven by the accumulation of metabolic risk factors in adulthood as opposed to pediatric reference ranges who have significantly lower ranges than the adult at base line. However, we would like to justify that in the context of Northern Ethiopia, this reverse trend is driven by unique epidemiological landscape of the area where there is early-life burden of Schistosomiasis and immune-active Hepatitis B infections in the pediatric population which can cause elevated ALT/or high viral loads. This explains why our community findings differ from the trends seen or metabolic driven studies.

Revisions in the manuscript: We have revised the discussion to address these discrepancies, as detailed in lines 381-387.

6. Reviewer’s comment: The description of the regression analysis lacks several important details. Although bivariate logistic regression followed by multivariable analysis was conducted, whether key confounders (age and sex) were considered for the adjustments.

Authors’ Response: We thank the reviewer for this important request for clarification. We agree that adjusting for fundamental demographic confounders is essential for the validity of the multivariable model. In our analysis, we followed a robust two-step approach to confounding. Initially, potential risk factors with a p-value <0.25 in the bivariate analysis were considered for multivariable analysis. Furthermore, we treated age and sex as a priori confounders to be forced into the final model regardless of their initial significance.

Revisions in the manuscript: We have updated the methods section under Data Analysis section (202-205) and the results section (lines 317-340) to explicitly state that age and sex were included in the multivariable model as fixed covariates to control for potential confounding.

7. Reviewer’s comment: The authors used bivariate logistic regression models with P < 0.25 in the bivariate analysis. Why was p value <0.25 selected? If p< 0.05 is considered, the conclusion will be different?

Authors’ Response: We appreciate the reviewer’s question regarding our selection of the p < 0.25 threshold for multivariable entry. The choice of p < 0.25 is a recognized strategy in purposeful selection of covariates, as recommended by Bursac et.al (2008) and Hosmer and Lemeshow (2000). Using a strict P<.05 cut-off in the bivariate analysis might fails to identify variables that are known to be important confounders or those that become significant only when adjusted for other covariates. By using a broader screening threshold (P<.25), we ensure that we do not prematurely exclude variables that contribute to a more stable and biologically plausible multivariable model.

8. Reviewer’s comment: The reported association between HBV infection and abnormal ALT (aOR ≈ 18) is extremely strong and requires careful interpretation.

Author’s Response: We thank the reviewer for highlighting this. Although the value of aOR has change a from 18 to 13 (after adjusting for age), we agree that such a large effect size warrants cautious interpretation and we have addressed it thoroughly in our revisions. The high association between HBV and elevated ALT underscores the potency of viral hepatitis as a driver of liver injury in this community. This magnitude is consistent with the natural history of HBV in endemic regions, where early-life infection often leads to prolonged immune-active phases characterized by intense hepatocellular damage and high transaminase levels. While the odds ratio is high, it highlights the urgent need for expanded screening and antiviral intervention to mitigate this profound risk.

Revisions in the manuscript: We have expanded the discussion (lines 395-399) to contextualize this finding against regional HBV epidemiology.

Minor comments (Reviewer 1)

1. The text indicates that most participants were from rural areas, while the table shows 67.5% of urban residence. This inconsistency must be corrected.

Response: We thank the reviewer for pointing out this discrepancy. There was a labeling error in the table where the ‘rural’ and ‘urban’ categories were misplaced. This has now been corrected in the revised table to ensure consistency with the text.

2. Albumin is reported as mg/dL, whereas the correct unit is g/dL. This error should be corrected throughout the manuscript.

Response: Thank you for the observation. The units for albumin have been corrected to g/dL throughout the revised manuscript to ensure clinical and scientific accuracy (revisions are shown).

3. Several minor errors are present. For examples, “Sever” → Severe, “Mini.” → Min, “73,5” → 73.5.

Response: We thank the reviewer for the careful proofreading. We have corrected the identified errors and have made a thorough review of the entire manuscript to correct any typographical errors.

Reviewer 2:

1. Reviewer’s comment: The major concern in the study is the single-time measurements of liver function biomarkers. Although the study covers a 5-year period liver function was assessed only at one time point this may capture transient abnormalities rather than chronic liver disease, and lead to misclassification of the liver health status of participants.

Authors’ Response: We sincerely thank the reviewer for identifying this discrepancy regarding the study period. We apologize for the confusion caused by the dates provided in the original abstract. To clarify, this research was conducted as part of a PhD study. While the entire project-including laboratory analysis, data entry, statistical processing, and write-up was extended from 2019 to 2024, the actual field data collection and specimen sampling occurred between December 2019 and June 2020. The study was intended to be a community-based cross-sectional study. We also agree that a single-point measurement reflects a snapshot of the population. We have now corrected the dates in the Abstract (line 33) and Methods section (120) to accurately reflect the 6 months data collection period (December 2019-June 2020) to ensure readers understand the cross-sectional nature of the data. It was also indicated as a limitation in the discussion section acknowledging that single-time measurements may capture transient elevations in biomarkers considering the cross-sectional nature of the study.

Revisions in the manuscript: revisions made in the abstract section line 33-34.

2. Reviewer’s Comment: Interpretation of liver health is based on analysis of each biomarker independently (AST/ALT ratios not computed).

Author’s Response: We would like to sincerely thank the reviewer for highlighting the importance of these parameters. In our initial analysis and earlier drafts, we had calculated the AST/ALT ratio (De Ritis ratio). However, we chose to omit these results from the previous version of the manuscript, as we initially felt they might serve as weaker independent indicators of liver injury compared to absolute biomarker values.

Upon re-evaluating the manuscript in light of your comments, we agree that the AST/ALT ratio (AAR) provides critical insights into the pattern of liver injury (distinguishing between alcoholic-hepatocellular with non-alcoholic like patterns) and adds significant value to the clinical interpretation of our findings. We have now incorporated these results into the manuscript. Besides, we have also incorporated additional points on R-value to determine the patterns of liver injury. We are grateful to the reviewer for noting the importance of these parameters, as we believe their inclusion has strengthened the paper.

Revisions in the manuscript: We have included the methods section, determination of patterns of liver injury sub-section, we have indicated the AAR ratio (170-172), and the R value on classification of liver injury patterns (line 173-177). In the results section, we have incorporated the AST/ALT ratio (AAR). Besides, the mean AAR, and category were calculated to indicate the etiologies of liver injury as indicated in lines 280-295, and the R-values to determine the patterns of liver injury (lines 296-315). We have also revised the discussion sections based on the AAR and R-value findings (436-455).

3. Comment: Wide age range. The study population varied from 5 to 80 years and raises concerns if mean values can be skewed by the effect of age.

Author Response: We thank the reviewer for highlighting regarding the broad age range i

Attachment Submitted filename: Response to Reviewers.docx

# Peer review: Decision Letter 1

**DOI:** 10.1371/journal.pone.0352266.r003

**Authors:** Seth Domfeh, Seth Domfeh

9 Jun 2026

<p>Biomarkers of chronic liver disease and their determinants in northern Ethiopia: Evaluating the synergistic impact of HBV and Schistosoma mansoni and the contribution of metabolic and lifestyle factors to liver injury .

PONE-D-26-09027R1

Dear Dr. Hailu,

We’re pleased to inform you that your manuscript has been judged scientifically suitable for publication and will be formally accepted for publication once it meets all outstanding technical requirements.

Within one week, you’ll receive an e-mail detailing the required amendments. When these have been addressed, you’ll receive a formal acceptance letter and your manuscript will be scheduled for publication.

An invoice will be generated when your article is formally accepted. Please note, if your institution has a publishing partnership with PLOS and your article meets the relevant criteria, all or part of your publication costs will be covered. Please make sure your user information is up-to-date by logging into Editorial Manager at Editorial Manager® and clicking the ‘Update My Information' link at the top of the page. For questions related to billing, please contact billing support.

If your institution or institutions have a press office, please notify them about your upcoming paper to help maximize its impact. If they’ll be preparing press materials, please inform our press team as soon as possible -- no later than 48 hours after receiving the formal acceptance. Your manuscript will remain under strict press embargo until 2 pm Eastern Time on the date of publication. For more information, please contact onepress@plos.org.

Kind regards,

Seth Agyei Domfeh, PhD

Academic Editor

PLOS One

Additional Editor Comments (optional):

Reviewers' comments:

Reviewer's Responses to Questions

**Comments to the Author**

1. If the authors have adequately addressed your comments raised in a previous round of review and you feel that this manuscript is now acceptable for publication, you may indicate that here to bypass the “Comments to the Author” section, enter your conflict of interest statement in the “Confidential to Editor” section, and submit your "Accept" recommendation.

Reviewer #2: All comments have been addressed

**********

2. Is the manuscript technically sound, and do the data support the conclusions?

The manuscript must describe a technically sound piece of scientific research with data that supports the conclusions. Experiments must have been conducted rigorously, with appropriate controls, replication, and sample sizes. The conclusions must be drawn appropriately based on the data presented.

Reviewer #2: Yes

**********

3. Has the statistical analysis been performed appropriately and rigorously?

Reviewer #2: (No Response)

**********

4. Have the authors made all data underlying the findings in their manuscript fully available?

The PLOS Data policy requires authors to make all data underlying the findings described in their manuscript fully available without restriction, with rare exception (please refer to the Data Availability Statement in the manuscript PDF file). The data should be provided as part of the manuscript or its supporting information, or deposited to a public repository. For example, in addition to summary statistics, the data points behind means, medians and variance measures should be available. If there are restrictions on publicly sharing data—e.g. participant privacy or use of data from a third party—those must be specified.

Reviewer #2: Yes

**********

5. Is the manuscript presented in an intelligible fashion and written in standard English?

PLOS ONE does not copyedit accepted manuscripts, so the language in submitted articles must be clear, correct, and unambiguous. Any typographical or grammatical errors should be corrected at revision, so please note any specific errors here.

Reviewer #2: Yes

**********

6. Review Comments to the Author

Please use the space provided to explain your answers to the questions above. You may also include additional comments for the author, including concerns about dual publication, research ethics, or publication ethics. (Please upload your review as an attachment if it exceeds 20,000 characters)

Reviewer #2: The authors have sufficiently addressed all concerns initially raised and significantly revised the manuscript to an acceptable level.

**********

7. PLOS authors have the option to publish the peer review history of their article (what does this mean?). If published, this will include your full peer review and any attached files.

If you choose “no”, your identity will remain anonymous but your review may still be made public.

**Do you want your identity to be public for this peer review?**   For information about this choice, including consent withdrawal, please see our Privacy Policy.

Reviewer #2: No

**********

# Editor report: Acceptance letter

**DOI:** 10.1371/journal.pone.0352266.r004

**Authors:** Seth Domfeh, Seth Domfeh

PONE-D-26-09027R1

PLOS One

Dear Dr. Hailu,

I'm pleased to inform you that your manuscript has been deemed suitable for publication in PLOS One. Congratulations! Your manuscript is now being handed over to our production team.

At this stage, our production department will prepare your paper for publication. This includes ensuring the following:

* All references, tables, and figures are properly cited

* All relevant supporting information is included in the manuscript submission,

* There are no issues that prevent the paper from being properly typeset

You will receive further instructions from the production team, including instructions on how to review your proof when it is ready. Please keep in mind that we are working through a large volume of accepted articles, so please give us a few days to review your paper and let you know the next and final steps.

Lastly, if your institution or institutions have a press office, please let them know about your upcoming paper now to help maximize its impact. If they'll be preparing press materials, please inform our press team within the next 48 hours. Your manuscript will remain under strict press embargo until 2 pm Eastern Time on the date of publication. For more information, please contact onepress@plos.org.

You will receive an invoice from PLOS for your publication fee after your manuscript has reached the completed accept phase. If you receive an email requesting payment before acceptance or for any other service, this may be a phishing scheme. Learn how to identify phishing emails and protect your accounts at https://explore.plos.org/phishing.

If we can help with anything else, please email us at customercare@plos.org.

Thank you for submitting your work to PLOS ONE and supporting open access.

Kind regards,

PLOS ONE Editorial Office Staff

on behalf of

Dr. Seth Agyei Domfeh

Academic Editor

PLOS One
